# Exploration of prognostic genes and risk signature in breast cancer patients based on RNA binding proteins associated with ferroptosis

**DOI:** 10.3389/fgene.2023.1025163

**Published:** 2023-02-24

**Authors:** Xiang Chen, Changcheng Yang, Wei Wang, Xionghui He, Hening Sun, Wenzhi Lyu, Kejian Zou, Shuo Fang, Zhijun Dai, Huaying Dong

**Affiliations:** ^1^ Department of General Surgery, Hainan General Hospital, Hainan Affiliated Hospital of Hainan Medical University, Haikou, China; ^2^ Department of Medical Oncology, The First Affiliated Hospital of Hainan Medical University, Haikou, China; ^3^ Department of Clinical Oncology, The University of Hong Kong, Hong Kong SAR, China; ^4^ Department of Oncology, The Seventh Affiliated Hospital, Sun Yat-sen University, Shenzhen, China; ^5^ Department of Breast Surgery, The First Affiliated Hospital, School of Medicine, Zhejiang University, Hangzhou, China

**Keywords:** breast cancer, ferroptosis, RNA-binding proteins, risk signature, bioinformatics analysis

## Abstract

**Background:** Breast cancer (BRCA) is a life-threatening malignancy in women with an unsatisfactory prognosis. The purpose of this study was to explore the prognostic biomarkers and a risk signature based on ferroptosis-related RNA-binding proteins (FR-RBPs).

**Methods:** FR-RBPs were identified using Spearman correlation analysis. Differentially expressed genes (DEGs) were identified by the “limma” R package. The univariate Cox and multivariate Cox analyses were executed to determine the prognostic genes. The risk signature was constructed and verified with the training set, testing set, and validation set. Mutation analysis, immune checkpoint expression analysis in high- and low-risk groups, and correlation between risk signature and chemotherapeutic agents were conducted using the “maftools” package, “ggplot2” package, and the CellMiner database respectively. The Human Protein Atlas (HPA) database was employed to confirm protein expression trends of prognostic genes in BRCA and normal tissues. The expression of prognostic genes in cell lines was verified by Real-time quantitative polymerase chain reaction (RT-qPCR). Kaplan-meier (KM) plotter database analysis was applied to predict the correlation between the expression levels of signature genes and survival statuses.

**Results:** Five prognostic genes (GSPT2, RNASE1, TIPARP, TSEN54, and SAMD4A) to construct an FR-RBPs-related risk signature were identified and the risk signature was validated by the International Cancer Genome Consortium (ICGC) cohort. Univariate and multivariate Cox regression analysis demonstrated the risk score was a robust independent prognostic factor in overall survival prediction. The Tumor Mutational Burden (TMB) analysis implied that the high- and low-risk groups responded differently to immunotherapy. Drug sensitivity analysis suggested that the risk signature may serve as a chemosensitivity predictor. The results of GSEA suggested that five prognostic genes might be related to DNA replication and the immune-related pathways. RT-qPCR results demonstrated that the expression trends of prognostic genes in cell lines were consistent with the results from public databases. KM plotter database analysis suggested that high expression levels of GSPT2, RNASE1, and SAMD4A contributed to poor prognoses.

**Conclusion:** In conclusion, this study identified the FR-RBPs-related prognostic genes and developed an FR-RBPs-related risk signature for the prognosis of BRCA, which will be of great significance in developing new therapeutic targets and prognostic molecular biomarkers for BRCA.

## Introduction

Breast cancer (BRCA) is one of the most common malignancies in women, accounting for a quarter of all female cancer cases (Siegel et al., 2020). One statistic shows that 2.26 million cases and 684,996 fatalities of BRCA were reported globally in 2020 (Sung et al., 2021). According to hormonal status, three different types of BRCA may be identified clinically: luminal-like, human epidermal growth factor receptor 2 (HER2) positive, and triple negative BRCA (TNBC) (Tian et al., 2021). Currently, BRCA treatment strategies mainly include the combination of surgical resection, endocrine therapy, chemotherapy, immunotherapy, and other adjuvant therapies (Waks and Winer, 2019). Despite tremendous improvements in early detection and treatment over the past few decades, the high morbidity and mortality of BRCA continue to constitute a serious danger to human health (Early Breast Cancer Trialists’ Collaborative Group (EBCTCG), 2011). Therefore, accurate prediction of BRCA prognosis is essential to improve prognosis and provide appropriate treatment for patients.

In contrast to cell necrosis, apoptosis, and autophagy, ferroptosis is a novel type of regulated cell death that is brought on by the accumulation of iron-dependent lipid peroxides (Song et al., 2021). Ferroptosis-related genes (FRGs) are promising therapeutic targets for BRCA patients (Li et al., 2021), and FRG signatures have been reported to be effective in predicting the prognosis of BRCA patients in earlier research (Liu et al., 2021). Some genes, such as ACSL4 and P53RRA, are known to positively regulate ferroptosis. However, other ferroptosis-related genes, including ATF2, NRF2, and GPX4, may inhibit the ferroptosis process in BRCA (Peng et al., 2021).

RNA binding proteins (RBPs) are proteins that interact with RNA through RNA binding domains. RBPs, as important coordinators for maintaining genome integrity, are widely expressed in cells and play a central role in gene regulation. RBPs are involved in regulating various aspects of RNA metabolism and function, including RNA biogenesis, maturation, transport, cell localization, and degradation, which have also been found to play a key role in tumor development (Luo et al., 2021). For example, RBP-related prognostic markers are highly expressed in BRCA (Lan et al., 2021), and TRIM21 facilitates the transformation of breast cancer cells from epithelium to stroma (Jin et al., 2019). In addition, RBPs can be used to predict the prognosis of patients with lung adenocarcinoma (Li et al., 2020a), and FOXK2 promotes colorectal cancer metastasis by up-regulating ZEB1 and EGFR expression (Du et al., 2019). However, no study has been undertaken on the prognostic significance of RBPs associated with ferroptosis in BRCA.

Therefore, bioinformatics methods were used to identify ferroptosis-related RBPs, establish an independent prognostic model, and verify the good predictive performance of the model. Further research was conducted on the variations in immune checkpoint expression, chemotherapeutic agent sensitivity, and TMB between high-risk and low-risk BRCA patients. The prognostic signature in this study may improve prognostic prediction and become the choice of treatment for patients with BRCA.

## Materials and methods

### Data source

RNA-seq data and clinical information of 1091 BRCA samples and 113 normal samples were accessed from the Cancer Genome Atlas (TCGA) database. RNA-seq data of 50 BRCA patients with survival information were collected from the ICGC database (https://dcc.icgc.org/) and utilized for risk signature validation, namely validation set. 117 BRCA samples from GSE88770 dataset were also used to verify the prognostic risk model. 259 ferroptosis-related genes (FRGs) were extracted from the FerrDb database. 1542 RNA-binding proteins (RBPs) were derived from a previous report (Wang et al., 2021).

### Authentication of differentially expressed genes (DEGs) in BRCA

The R language package ‘limma’ was engaged in analyzing the DEGs between 113 normal samples and 1091 BRCA samples from the TCGA database (Ritchie et al., 2015). The screening criteria for difference were |log_2_Fold change (FC)| > 1 and adjusted *p*-value <0.05.

### Excavation of FR-RBP genes in BRCA

The expression matrices of FRGs and RBPs were extracted sequentially based on the transcriptome data from the TCGA database, followed by the calculation of correlation coefficients and *p*-values between FRGs and RBPs using the Spearman method. The RBPs were identified as significantly correlated with FRG, i.e., FR RBPs, by filtering with a threshold FDR <0.05 and |correlation coefficient (cor)| > 0.3. The function of differentially expressed FR-RBPs (DE-FR-RBPs) was probed by the R package ‘clusterProfiler’ (Yu et al., 2012). Gene Ontology (GO) enrichment analysis mainly described the biological processes (BP), cellular components (CC), and molecular functions (MF) correlated with genes. The Kyoto Encyclopedia of Genes and Genomes (KEGG) pathway enrichment analysis revealed biological pathways associated with genes.

### Establishment and validation of the risk signature based on DE-FR-RBPs for BRCA

To estimate whether the DE-FR-RBPs were associated with survival in BRCA patients, we randomly split the 1069 patients with survival information from the TCGA database into a training set and a testing set in a 6:4 ratio, and the clinicopathologic characteristics of the training set and testing set are shown in Table 1. Univariate Cox and multivariate Cox regression analyses were executed in the training set to screen prognostic genes. The risk score was calculated as Risk score = h0(t)*exp (β_1_X_1_ +β_2_X_2_+…+β_n_X_n_), where *β* refers to the regression coefficient; X represented the gene expression level; h0(t) is the benchmark risk function. The following steps were performed simultaneously in the training set, testing set, validation set, and GSE88770 dataset. We calculated risk scores for each BRCA patient based on prognostic genes and regression coefficients and divided BRCA patients into high-risk and low-risk groups based on the optimal cutoff values obtained from the surv_cutpoint function in the ‘survival’ package. A Kaplan-Meier (K-M) curve, a risk curve, and a prognostic gene expression heatmap were plotted, and receiver operating characteristic (ROC) curves for 1,3,5-year survival were also drawn to assess the accuracy of the risk signature in predicting survival.

**TABLE 1:** The clinicopathologic characteristics of training set and testing set.

Features	TCGA	
	Training set (*n* = 642)	testing set (*n* = 427)
age (%)		
≤30	7 (1.1)	5 (1.2)
>30	635 (98.9)	422 (98.8)
pathologic_M (%)		
M0	535 (83.3)	355 (83.1)
M1	13 (2.0)	9 (2.1)
MX	94 (14.6)	63 (14.8)
pathologic_N (%)		
N0	310 (48.3)	192 (45.0)
N1	207 (32.2)	150 (35.1)
N2	67 (10.4)	53 (12.4)
N3	47 (7.3)	26 (6.1)
NX	11 (1.7)	6 (1.4)
pathologic_T (%)		
T1	179 (27.9)	100 (23.4)
T2	361 (56.2)	256 (60.0)
T3	75 (11.7)	57 (13.3)
T4	25 (3.9)	13 (3.0)
TX	2 (0.3)	1 (0.2)
tumor_stage.diagnoses (%)		
stage I	113 (17.6)	68 (15.9)
stage II	360 (56.1)	246 (57.6)
stage III	145 (22.6)	95 (22.2)
stage IV	12 (1.9)	8 (1.9)
stage x	6 (0.9)	5 (1.2)
Unknow	6 (0.9)	5 (1.2)
race.demographic (%)		
american indian or alaska native	1 (0.2)	0 (0.0)
asian	37 (5.8)	21 (4.9)
black or african american	107 (16.7)	73 (17.1)
Unknow	52 (8.1)	33 (7.7)
white	445 (69.3)	300 (70.3)

### Establishment of a nomogram

Chi-square tests were applied to examine the linkage of risk signatures with other clinicopathological characteristics. Independent prognostic profiling was undertaken by adopting univariate Cox and multivariate Cox analysis. Clinical features and risk scores were selected to establish a nomogram associated with outcome for assessing the overall survival (OS) of 1-, 3-, and 5-year for BRCA patients. Furthermore, calibration curve was plotted to evaluate the consistency between predicted probabilities of 1-, 3- and 5-year survival and actual ones.

### Analysis based on risk signature

To investigate the relevance of risk signature to somatic mutations, immune checkpoints, immune cell infiltration, and chemotherapeutic efficacy, we conducted mutation analysis, immune checkpoint expression analysis, and immune cell infiltration analysis in high- and low-risk groups, and discovered the correlation between risk signature and chemotherapeutic agents using the “maftools” package, the “ggplot2” package, CIBERSORT algorithm and the CellMiner database respectively.

### Analysis of prognostic genes

We used the cBioPortal database (http://www.cbioportal.org) to analyze genetic alterations of prognostic genes in risk signatures. The HPA database (https://www.proteinatlas.org/) was employed to identify protein expression trends of prognostic genes in BRCA and normal tissues. To further explore the possible molecular mechanisms of prognostic genes in BRCA, we performed a single-gene Gene Set Enrichment Analysis (GSEA) analysis based on the KEGG gene set, setting SIZE >20, NOM *p*-value <0.05, and FDR q-val >0.05 as significant pathways.

### Verification of mRNA expression levels of prognostic genes in cell lines

Human epithelial cell lines from the mammary gland, MCF-10A, and three breast cancer cell lines MCF-7, MDA-MB-468, and T47D were obtained from iCell Bioscience Inc. (Shanghai, China). MCF-10A cells were cultured in MEGM Kit medium (Lonza/Clonetics, CC-3150). MDA-MB-468 and T47D cells were cultured in RPMI-1640 medium (iCell-0002), supplemented with 0.02 mg/L of bovine insulin (iCell-0016-a), 10% fetal bovine serum (FBS) (Gibco) and 1% penicillin/streptomycin. MCF-7 cells were cultured in MEM basic medium (iCell-0012), supplemented with 0.01 mg/mL of bovine insulin, 10% FBS and 1% penicillin/streptomycin. The cells were incubated at 37°C in a humidified atmosphere of 5% CO_2_. Total RNA from the cell lines in logarithmic phage was isolated utilizing the TRIzol Reagent following the producer’s instructions (Ambion, USA). Next, total RNA was reverse transcribed into cDNA utilizing the SweScript-First-strand-cDNA-synthesis-kit (Servicebio, China) and qPCR was subsequently carried out using the 2xUniversal Blue SYBR Green qPCR Master Mix, according to the manufacturers’ direction (Servicebio, China). The sequences of the primers were listed in Table 2. The relative expression level was normalized to the endogenous control GAPDH and calculated using the 2^−ΔΔCq^ method (Livak and Schmittgen, 2001). The Student’s t-test was used to contrast the distinction. The two-tailed *p*-value <0.05 was delimited as statistically significant.

**TABLE 2:** The sequences of the primers for qPCR.

Primer	Sequences
**GSPT2 F**or	CGTCAACGCCAAGCC
**GSPT2** Rev	CCCCCGTCCCATCCT
**RNASE1 F**or	ACTCAGACAGTTCCCCCA
**RNASE1 R**ev	CCTCCACAGAAGCATCAA
**SAMD4A F**or	AAC​CAA​TGG​CAA​CAG​GAA​T
**SAMD4A R**ev	GGT​GGG​GAC​AGA​TGA​GGA​G
**TIPARP F**or	GGC​AGA​TCA​AAA​GGA​CAA​C
**TIPARP R**ev	ATA​AAA​CAG​GAG​CGG​AAG​A
**TSEN54 F**or	AAG​AAT​AAC​CCT​GGC​AAA​C
**TSEN54 R**ev	AAG​TCC​CTG​AAG​CTG​TAG​A
**GAPDH F**or	CCC​ATC​ACC​ATC​TTC​CAG​G
**GAPDH R**ev	CAT​CAC​GCC​ACA​GTT​TCC​C

### The Kaplan-Meier (KM) plotter for prognostic value

The survival probability of signature mRNA expression was assessed using the KM plot database (www.kmplot.com), which contained survival information and gene expression data for BRCA patients. In order to analyze the OS of BRCA patients, the samples were divided into high- and low-expression groups by median expression and evaluated by a KM survival plot, with the log-rank *p*-value and hazard ratio (HR) with 95% confidence intervals (CIs).

### Statistical analysis

All bioinformatics analyzes were run in the R programming language and the data from different groups were compared using the Wilcoxon test. *p* values less than 0.05 were considered statistically significant if not specified above.

## Results

### DE-FR-RBPs in BRCA

**FIGURE 1:**
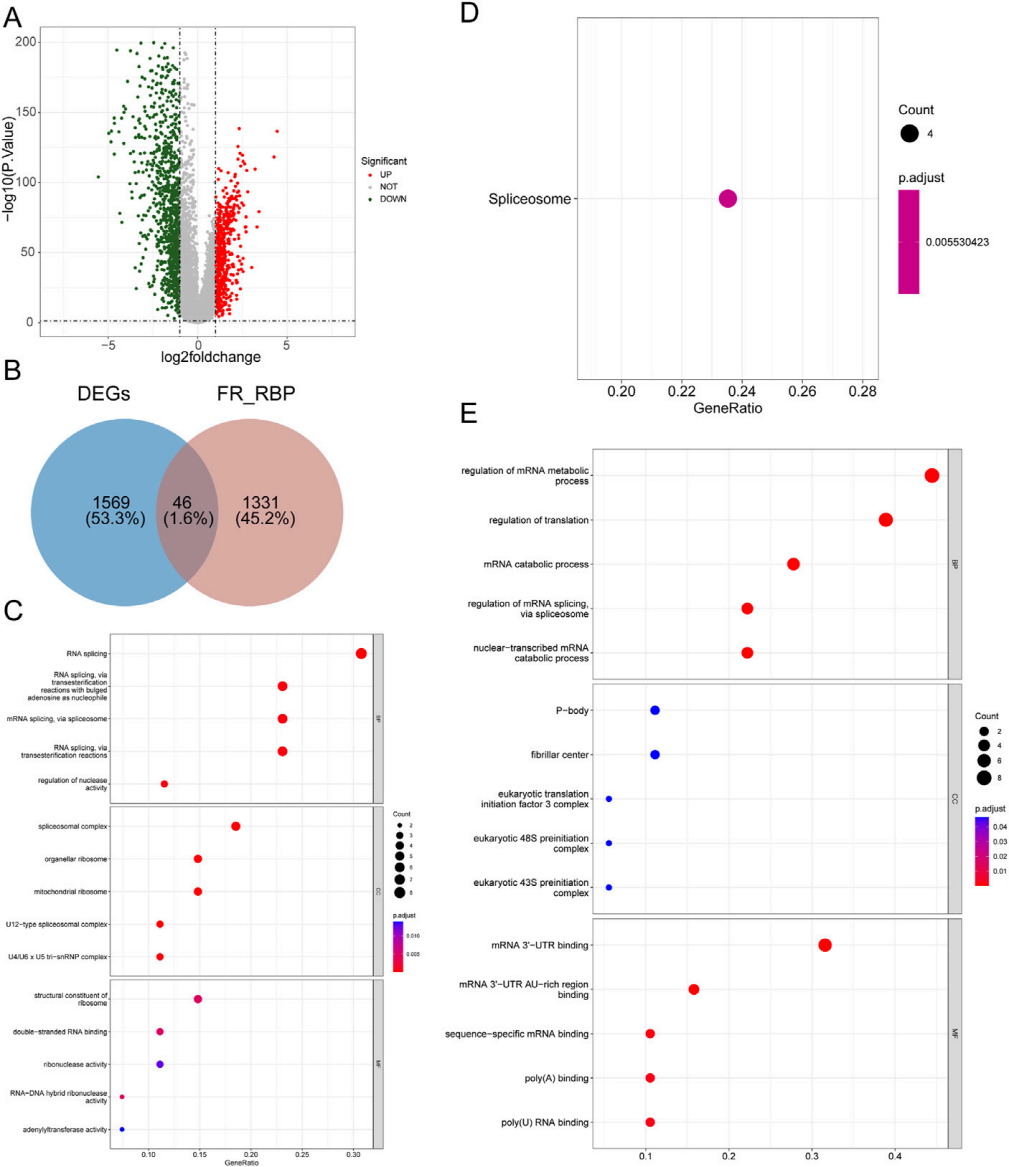
Identification of 46 DE-FR-RBPs and its functional enrichment analysis. **(A)** Volcano map of DEGs between BRCA and normal samples. Green dots represent downregulated genes while red dots indicate upregulated genes. **(B)** Venn diagram was conducted to detect the intersection of 46 DE-FR-RBPs by DEGs and FR-RBPs. **(C)** The top5 GO entries under each category based on the function enrichment results of upregulated DE-FR-RBPs. **(D)** The KEGG pathway enriched by upregulated DE-FR-RBPs. **(E)** The top5 GO entries under each category based on the function enrichment results of downregulated DE-FR-RBPs. The larger the circle contains the greater the number of genes and the redder the color the smaller the *p*-value.

As shown in Figure 1A and Supplementary Table S1, a total of 1615 DEGs, including 600 upregulated genes and 1015 downregulated genes, were exploited in BRCA samples compared with normal samples. The expression heatmaps of the top 50 upregulated genes and top 50 downregulated genes are displayed in Supplementary Figure S1A. Then, 1377 FR-RBPs were identified by Spearman correlation analysis between FRGs and RBPs (Supplementary Tables S2, S3). After overlapping the DEGs and FR-RBPs, 46 DE-FR-RBPs in BRCA, including 27 upregulated genes and 19 downregulated genes, were determined and all DE-FR-RBPs were RBPs (Figure 1B; Supplementary Figure 1B; Supplementary Table S4). To probe the possible functions of these 46 genes in BRCA development, we proceeded with a functional enrichment analysis. A total of 116 GO entries (56 BP, 47 CC, and 13 MF) and 1 KEGG pathway were enriched for upregulated genes (Supplementary Table S5). The downregulated genes were enriched for a total of 87 GO entries (59 BP, 8 CC, and 20 MF) (Supplementary Table S5). The top 5 entries in each category are shown in the bubble diagram (Figures 1C–E). The upregulated genes in KEGG pathways were associated with the “Spliceosome” pathway (Figure 1D). The majority of the upregulated genes in BP were connected to biological processes that included RNA splicing and regulation of nuclease activity (Figure 1C). Most of the downregulated genes were involved in the regulation of mRNA metabolism, translation, mRNA catabolism, mRNA splicing *via* the spliceosome, and nuclear-transcribed mRNA catabolism (Figure 1D).

### The risk signature based on the DE-FR-RBPs in BRCA

**FIGURE 2:**
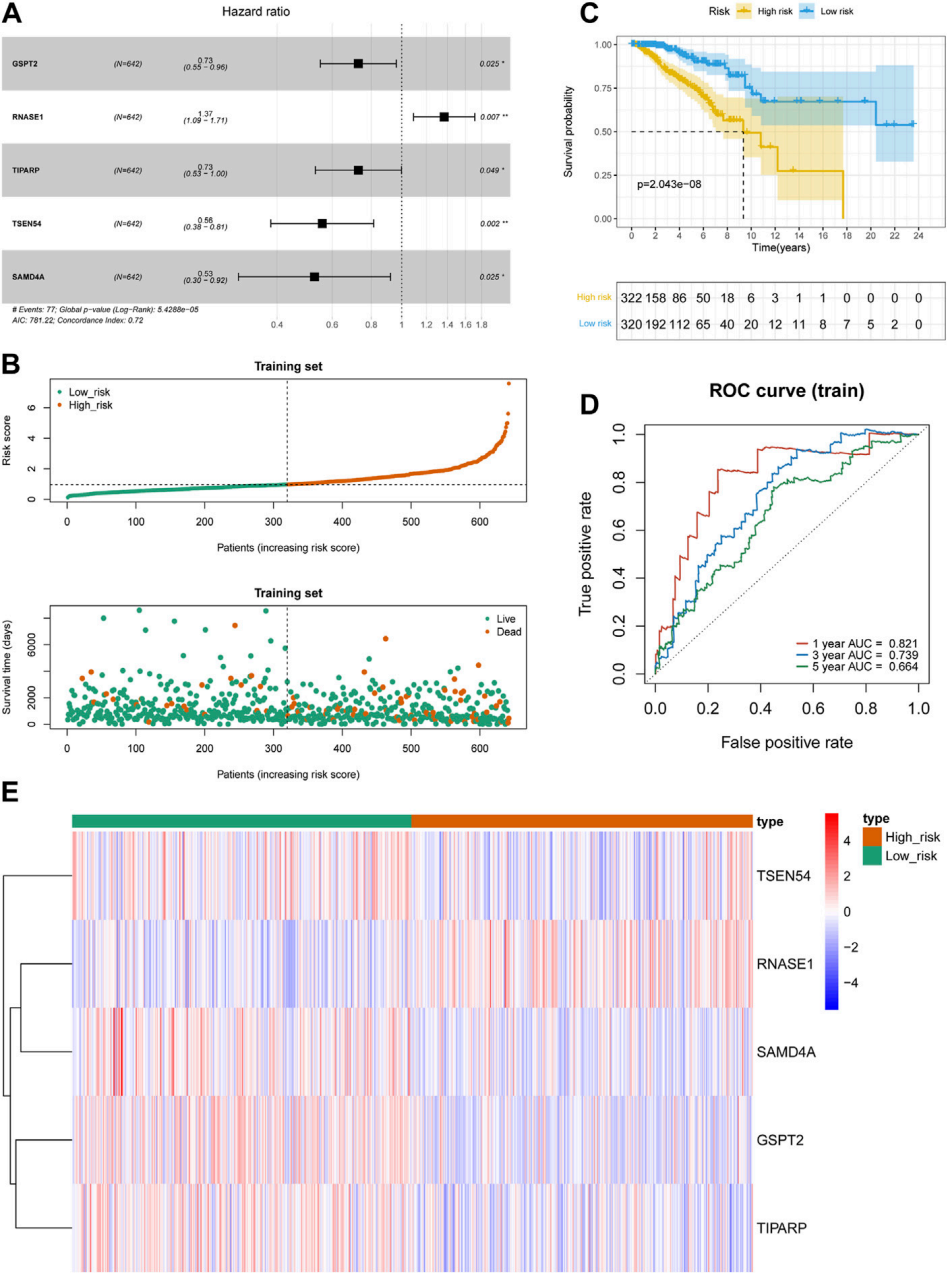
Construction of prognostic risk models in BRCA. **(A)** The forest map of multivariate Cox regression analysis. **(B)** Distribution of survival status based on the median risk score in training set. **(C)** Kaplan-Meier survival analysis of high and low risk groups in TCGA-BRCA training set. **(D)** Time-independent receiver operating characteristic (ROC) analysis of risk scores predicting the overall survival of 1-, 3-, and 5-year in training set. **(E)** Heatmap indicated the difference of prognostic gene expression between high- and low-risk groups in TCGA-BRCA training set.

We randomly assigned 1069 BRCA cases with survival information from the TCGA database into a training set and a testing set. We included 46 DE-FR-RBPs in a univariate Cox analysis in the training set. Based on the *p*-value <0.2, eight genes (SAMD4A, CELF2, TSEN54, ZFP36L2, TIPARP, GSPT2, YBX3, and RNASE1) were selected for incorporation into the next step of multivariate Cox analysis (Table 3). Five genes associated with overall survival (OS) in BRCA patients were screened (GSPT2, RNASE1, TIPARP, TSEN54, and SAMD4A) by multivariate Cox analysis for risk signature establishment (Figure 2A). Of these, GSPT2, TIPARP, TSEN54, and SAMD4A were protective factors for patient OS (hazard ratio (HR) < 1), and RNASE1 was a risk factor (HR > 1). The risk score was calculated as: risk score = h0(t)×exp ((-0.31839)×expression of GSPT2 + (0.312671)×expression of RNASE1 + (−0.31809)×expression of TIPARP + (−0.58387)×expression of TSEN54 + (−0.64123)×expression of SAMD4A). Based on this formula, we calculated the risk score for each BRCA patient in the training set and classified them into high- and low-risk groups based on optimal cut-off value (Figure 2B). The risk curve manifested that as the risk score increased, patients confronted a higher risk of demise (Figure 2B). The K-M curve indicated that the high-risk patients had noteworthy poorer survival than the low-risk patients (Figure 2C). The Area Under Curve (AUC) values for the ROC curves at 1, 3, and 5 years were 0.821, 0.739, and 0.664, reflecting the decent accuracy of the gene signature (Figure 2D; Supplementary Tables S6–S8). The heatmap revealed that RNASE1 was highly expressed in the high-risk group and GSPT2, TIPARP, TSEN54, and SAMD4A were highly expressed in the low-risk group (Figure 2E). To further confirm the applicability and reliability of the risk signature, the above analysis was carried out in both the testing set and the external validation set. The results of the testing set and external validation set were consistent with the training set (Supplementary Figures S2A–H). The AUC values of the 1, 3, and 5-year ROC curves in the testing set were 0.603, 0.649, and 0.606 respectively (Supplementary Figure S2C), while the AUC values of the 1, 3, and 5-year ROC curves in the validation set were 0.897, 0.978, and 0.846 respectively (Supplementary Figure S2G), suggesting that the FR-RBPs associated risk signature was an effective predictor of survival in BRCA patients. Moreover, the GSE88770 dataset was also used to validate the prognostic model (Supplementary Figures S2I–L). The results suggested that patients in high-risk group had poorer OS than low-risk group (Supplementary Figure S2J), and the AUC values of 3 and 5 years were 0.724 and 0.690 respectively (Supplementary Figure S2K), indicating the risk signature had good predictive ability.

**TABLE 3:** The result of Univariate Cox analysis.

id	HR	HR.95L	HR.95H	P-value
**GSPT2**	0.707641508	0.54167322	0.924462362	0.011214893
**RNASE1**	1.250347564	1.009428054	1.548767171	0.040766883
**TIPARP**	0.733565395	0.541961869	0.992907839	0.044851373
**TSEN54**	0.689105896	0.476337238	0.996913319	0.048112608
**SAMD4A**	0.662677227	0.406480148	1.080350687	0.098935143
**YBX3**	0.847845556	0.682996077	1.052483479	0.134586015
**CELF2**	0.828334478	0.630036769	1.089044389	0.177340052
**ZFP36L2**	0.844579069	0.659786412	1.081128364	0.179988295
**ALYREF**	0.821438156	0.606253705	1.113000447	0.204378613
**LARP6**	0.768080806	0.504944647	1.168342169	0.217593842
**NUDT16L1**	0.82550748	0.59655413	1.142331542	0.247259363
**EZH2**	0.867042096	0.65859947	1.141455515	0.309192942
**DDX39A**	0.86271146	0.630696363	1.180078255	0.355504011
**EXOSC4**	1.135808917	0.866411329	1.488971638	0.356588365
**BOP1**	1.116973789	0.878760146	1.419762208	0.366043977
**EXO1**	1.099478609	0.867536602	1.393431943	0.432744694
**QKI**	0.883711263	0.610381308	1.279438911	0.512607185
**ACO1**	0.87859728	0.595336873	1.296632571	0.514537779
**SNRPE**	1.136400042	0.773123506	1.670373553	0.515284169
**SNRPB**	0.889632146	0.622585703	1.271223143	0.520754293
**RBMS3**	0.898135035	0.624364616	1.29194788	0.562492321
**MAZ**	1.119591522	0.759603203	1.650184163	0.568172367
**LSM4**	0.900036413	0.621296936	1.303829936	0.577555478
**MRPL12**	0.933092038	0.730247914	1.192281053	0.579763207
**ZNF106**	1.101633388	0.760767574	1.595225878	0.608349773
**RBMS2**	0.886316661	0.556348736	1.411987074	0.611505345
**ZFP36**	0.951432459	0.781604851	1.15816032	0.619691033
**OAS3**	1.046277912	0.861897572	1.270101582	0.647397407
**EIF3L**	0.941677939	0.696351628	1.273433284	0.696358388
**ESRP1**	1.062783087	0.762096148	1.482106809	0.719706896
**OAS2**	1.029753483	0.873416765	1.214073598	0.727098229
**PARP1**	0.937945972	0.64125593	1.37190567	0.741252895
**RNASEH2A**	1.055259928	0.748967479	1.486811572	0.758473021
**ZCCHC24**	0.962498613	0.746990713	1.240180854	0.767576629
**NOVA1**	1.027431906	0.856821179	1.232014739	0.770212969
**SNRNP25**	0.950064372	0.663923509	1.359527564	0.779352003
**MBNL2**	0.967323505	0.723614741	1.293111805	0.822507597
**RNASE7**	1.063113031	0.603951646	1.871357292	0.832004214
**EEF1A2**	1.010017226	0.91721685	1.112206778	0.83937158
**HNRNPAB**	1.030484411	0.652800239	1.626681577	0.897417351
**OASL**	0.990460197	0.831607532	1.179656705	0.914412957
**RPUSD1**	0.986777119	0.681941484	1.427877764	0.943710688
**MRPS34**	0.99432936	0.728127266	1.357854487	0.971465208
**MEX3A**	0.99898961	0.830181567	1.202122862	0.991459526
**MRPL14**	1.001139283	0.708039533	1.415570483	0.994859575
**MRPS12**	1.000436745	0.7027528	1.424218703	0.998066621

### The FR-RBPs associated risk signature was an independent prognostic factor

**FIGURE 3:**
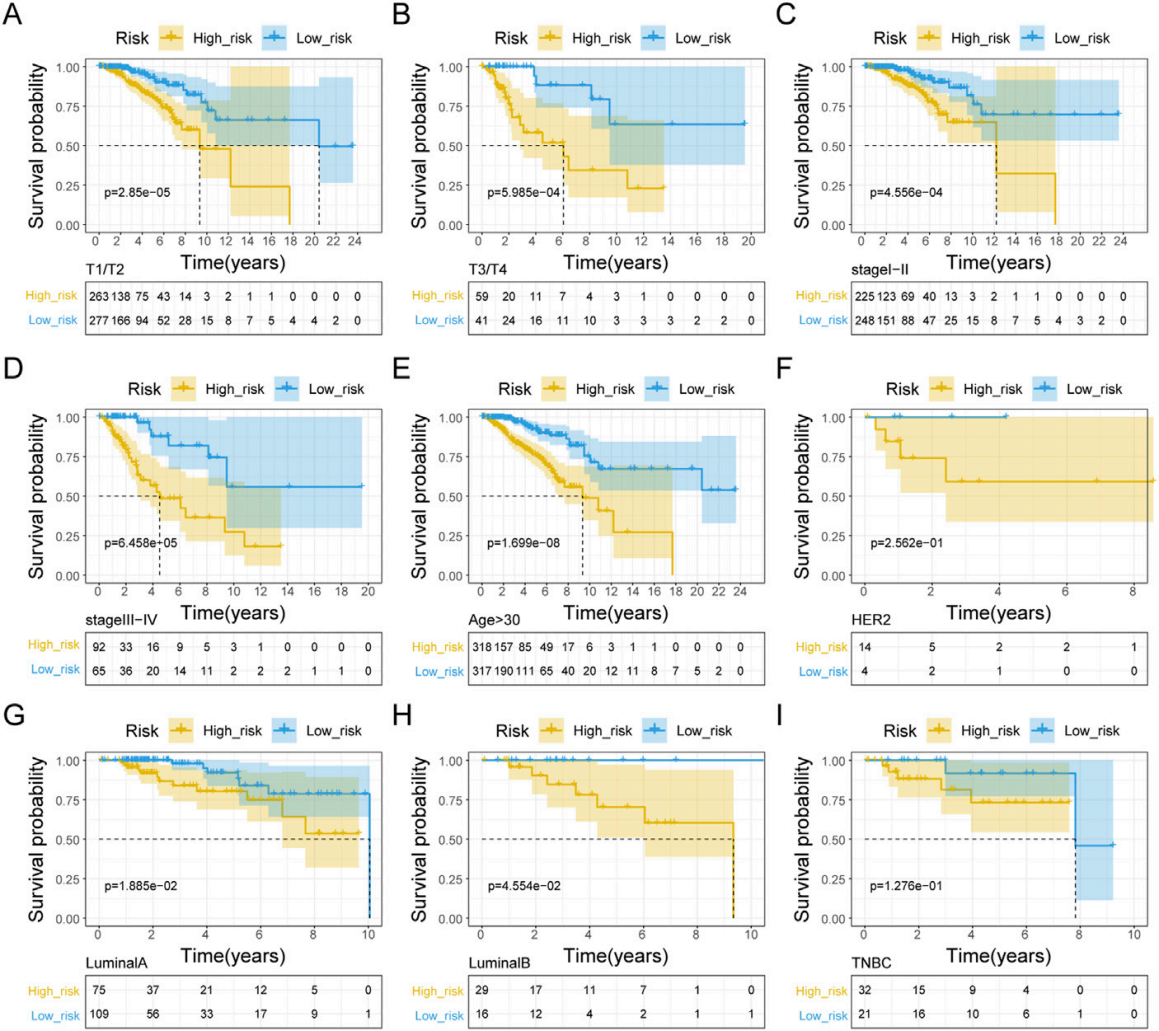
Kaplan-Meier curve analysis of clinical pathologic factors for the OS in BRAC patients in the training set. Stratified K-M curves among different groups, including pathologic T stage **(A, B)**, pathologic stage **(C, D)**, age **(E)**, HER2-enriched **(F)**, Luminal A **(G)**, Luminal B **(H)**, and TNBC **(I)**.

**FIGURE 4:**
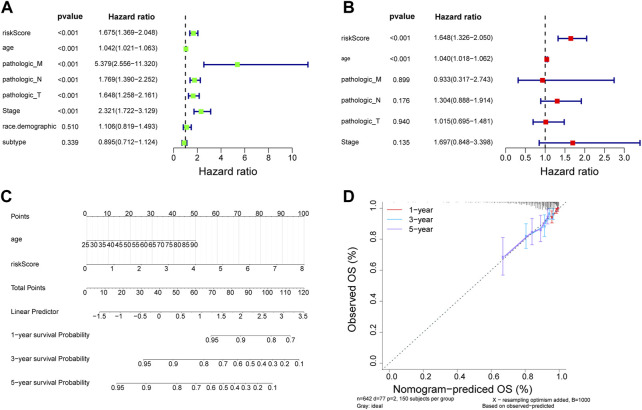
Estableshment of a nomogram. **(A)** Univariate Cox analysis of the correlations between risk score and clinicopathological factors in TCGA-BRCA training set. **(B)** Multivariate Cox of the correlations betweent the risk score and clinicopathological factors independent prognostic analysis of in TCGA-BRCA training set. **(C)** Nomogram for forecasting 1-, 3- and 5-year OS. **(D)** The calibration for forecasting 1-, 3- and 5-year OS.

We initially evaluated the proportion of individuals at high- and low-risk under various clinical features in order to better investigate the relationship between risk signature and clinicopathological factors. As shown in Table 4, risk signature was associated with pathologic T and stage in the training set, while in the testing set, risk signature was correlated with pathologic T (Table 5). Age and stage were unrelated to the risk signature in the validation set (Table 6). Stratified survival analysis showed significant differences in survival between high- and low-risk groups with different T subgroups, stage subgroups, age (age >30), and subtypes (HER2-enriched, LuminalA, LuminalB, and triple negative breast cancer (TNBC)) in the training set (Figures 3A–I). Next, we integrated both risk score and clinical factors into a univariate Cox analysis, with risk score, age, pathologic N, pathologic T, pathological M, and the stage being associated with OS in BRCA patients (Figure 4A). Then included these factors into multivariate Cox analysis, the results indicated that risk score and age were independent prognostic factors (Figure 4B).

**TABLE 4:** The number of patients in high- and low-risk groups with different clinical characteristics in the training set.

	TCGA training set			
	Total (*n* = 642)	High_risk (*n* = 322)	Low_risk (*n* = 320)	*p*-value
Age (years)				
**≤30**	7 (1.1%)	4 (1.2%)	3 (0.9%)	1
**>30**	635 (98.9%)	318 (98.8%)	317 (99.1%)	
pathologic_M				
**M0**	535 (83.3%)	269 (83.5%)	266 (83.1%)	0.314
**M1**	13 (2.0%)	9 (2.8%)	4 (1.3%)	
**MX**	94 (14.6%)	44 (13.7%)	50 (15.6%)	
pathologic_N				
**N0**	310 (48.3%)	156 (48.4%)	154 (48.1%)	0.298
**N1**	207 (32.2%)	94 (29.2%)	113 (35.3%)	
**N2**	67 (10.4%)	40 (12.4%)	27 (8.4%)	
**N3**	47 (7.3%)	26 (8.1%)	21 (6.6%)	
**NX**	11 (1.7%)	6 (1.9%)	5 (1.6%)	
pathologic_T				
**T1**	179 (27.9%)	74 (23.0%)	105 (32.8%)	0.0042
**T2**	361 (56.2%)	189 (58.7%)	172 (53.8%)	
**T3**	75 (11.7%)	40 (12.4%)	35 (10.9%)	
**T4**	25 (3.9%)	19 (5.9%)	6 (1.9%)	
**TX**	2 (0.3%)	0 (0%)	2 (0.6%)	
Stage				
**stage I**	113 (17.6%)	46 (14.3%)	67 (20.9%)	0.0209
**stage II**	360 (56.1%)	179 (55.6%)	181 (56.6%)	
**stage III**	145 (22.6%)	83 (25.8%)	62 (19.4%)	
**stage IV**	12 (1.9%)	9 (2.8%)	3 (0.9%)	
**stage x**	6 (0.9%)	1 (0.3%)	5 (1.6%)	
**Unknow**	6 (0.9%)	4 (1.2%)	2 (0.6%)	
race				
**american indian or alaska native**	1 (0.2%)	1 (0.3%)	0 (0%)	0.306
**asian**	37 (5.8%)	20 (6.2%)	17 (5.3%)	
**black or african american**	107 (16.7%)	49 (15.2%)	58 (18.1%)	
**Unknow**	52 (8.1%)	32 (9.9%)	20 (6.3%)	
**white**	445 (69.3%)	220 (68.3%)	225 (70.3%)	

**TABLE 5:** The number of patients in high- and low-risk groups with different clinical characteristics in the testing set.

	TCGA testing set			
	Total (*n* = 427)	High_risk (*n* = 99)	Low_risk (*n* = 328)	*p*-value
**Age (years)**				
**≤30**	5 (1.2%)	0 (0%)	5 (1.5%)	0.482
**>30**	422 (98.8%)	99 (100%)	323 (98.5%)	
pathologic_M				
**M0**	355 (83.1%)	83 (83.8%)	272 (82.9%)	0.235
**M1**	9 (2.1%)	4 (4.0%)	5 (1.5%)	
**MX**	63 (14.8%)	12 (12.1%)	51 (15.5%)	
pathologic_N				
**N0**	192 (45.0%)	36 (36.4%)	156 (47.6%)	0.114
**N1**	150 (35.1%)	35 (35.4%)	115 (35.1%)	
**N2**	53 (12.4%)	19 (19.2%)	34 (10.4%)	
**N3**	26 (6.1%)	7 (7.1%)	19 (5.8%)	
**NX**	6 (1.4%)	2 (2.0%)	4 (1.2%)	
pathologic_T				
**T1**	100 (23.4%)	15 (15.2%)	85 (25.9%)	0.0197
**T2**	256 (60.0%)	69 (69.7%)	187 (57.0%)	
**T3**	57 (13.3%)	9 (9.1%)	48 (14.6%)	
**T4**	13 (3.0%)	6 (6.1%)	7 (2.1%)	
**TX**	1 (0.2%)	0 (0%)	1 (0.3%)	
Stage				
**stage I**	68 (15.9%)	10 (10.1%)	58 (17.7%)	0.104
**stage II**	246 (57.6%)	54 (54.5%)	192 (58.5%)	
**stage III**	95 (22.2%)	29 (29.3%)	66 (20.1%)	
**stage IV**	8 (1.9%)	4 (4.0%)	4 (1.2%)	
**stage x**	5 (1.2%)	1 (1.0%)	4 (1.2%)	
**Unknow**	5 (1.2%)	1 (1.0%)	4 (1.2%)	
race				
**asian**	21 (4.9%)	2 (2.0%)	19 (5.8%)	0.259
**black or african american**	73 (17.1%)	14 (14.1%)	59 (18.0%)	
**Unknow**	33 (7.7%)	10 (10.1%)	23 (7.0%)	
**white**	300 (70.3%)	73 (73.7%)	227 (69.2%)	

**TABLE 6:** The number of patients in high- and low-risk groups with different clinical characteristics in the validation set.

	ICGC			
	Total (*n* = 50)	High_risk (*n* = 10)	Low_risk (*n* = 40)	*p*-value
**Age (years)**				
**≤30**	13 (26.0%)	5 (50.0%)	8 (20.0%)	0.126
**>30**	37 (74.0%)	5 (50.0%)	32 (80.0%)	
Stage				
**0**	3 (6.0%)	0 (0%)	3 (7.5%)	0.402
**1**	13 (26.0%)	4 (40.0%)	9 (22.5%)	
**2**	28 (56.0%)	4 (40.0%)	24 (60.0%)	
**3**	6 (12.0%)	2 (20.0%)	4 (10.0%)	

### Nomogram construction is based on risk scores and clinicopathological factors

Independent prognostic indicators including risk score and age were involved in the nomogram to forecast the survival probability of BRCA patients. Nomography predicted the 1, 3, and 5 years survival probability of patients with BRCA (Figure 4C). The calibration curve was plotted to assess the nomogram and indicated that the practical survival of BRCA patients was in line with the predicted value (Figure 4D).

### The role of risk signature in BRCA

**FIGURE 5:**
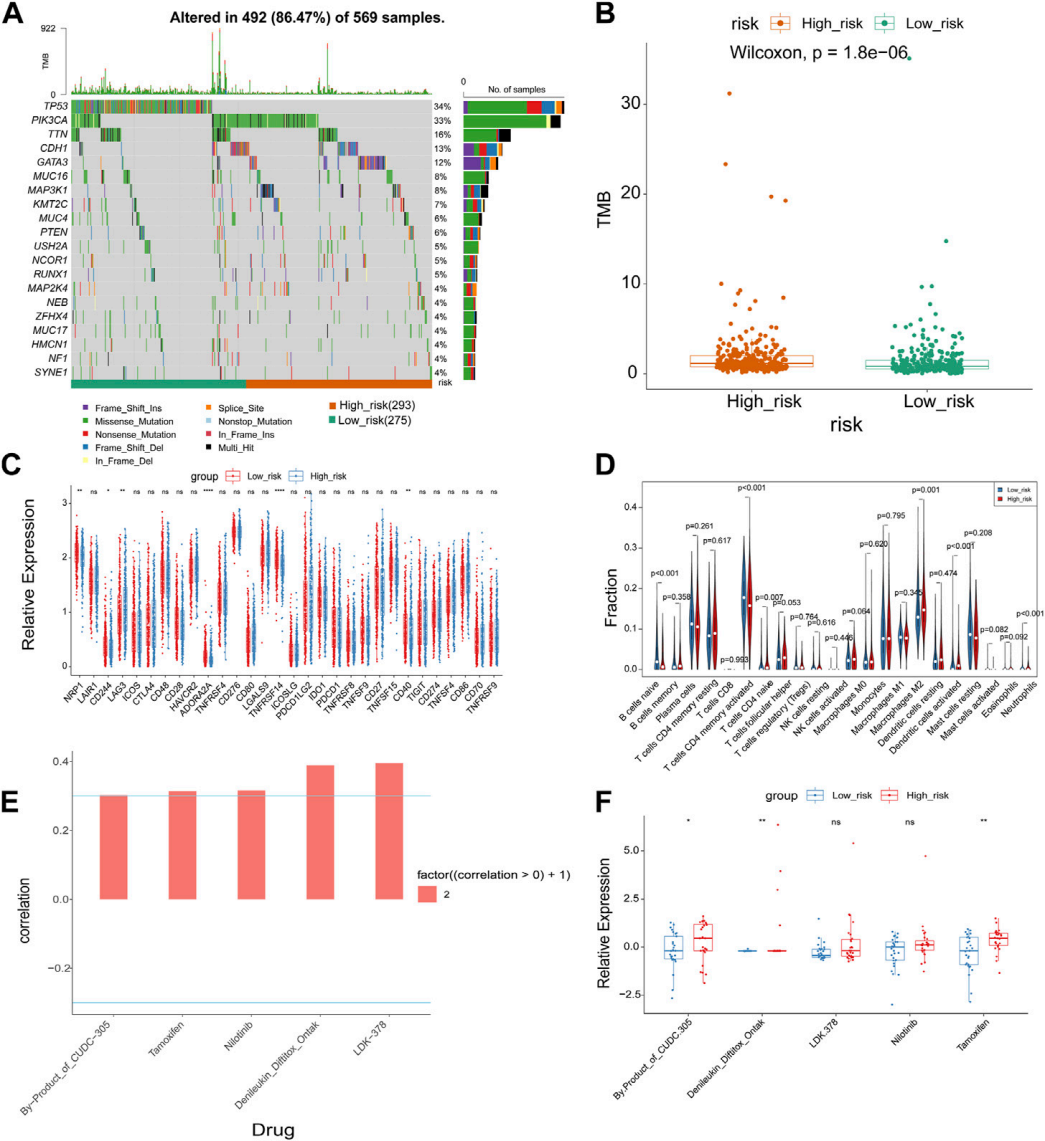
Correlation analysis of risk model with genomic mutations, immune checkpoints, immune cell infiltration, and chemotherapeutic drug efficacy. **(A)** The waterfall maps of the somatic mutations of the top 20 mutated genes in the high and low risk groups. **(B)** Boxplot of TMB score in high and low risk group. **(C)** Boxplot of the relationships of immune checkpoint expression between high and low risk group. **(D)** The immune cell infiltration using CIBERSORT. **(E)** Correlation analysis between drugs and risk score. **(F)** Boxplot of IC50 values of significantly related drugs in high and low risk group. *p-value <0.05, **p-value <0.01, ***p-value <0.001, ****p-value <0.0001.

We used the “maftools” program to display the distribution of the top 20 mutated genes in the high- and low-risk groups in order to further analyze the difference in mutations between the groups, revealing that TP53 was more frequently mutated in the low-risk group (Figure 5A). We further examined the tumor mutational burden (TMB) of high- and low-risk patients and noted that the TMB of the high-risk group was significantly higher than that of the low-risk group (Figure 5B). The TMB was associated with immunotherapy, implying that the high- and low-risk groups responded differently to immunotherapy. Following that, we compared the expression of immune checkpoint molecules in the high- and low-risk groups, displaying that NRP1, CD244, ADORA2A, TNFRSF14, and TNFRSF15 were significantly less expressed in the high-risk group than in the low-risk group and that LAG3 was significantly more expressed in the high-risk group than in the low-risk group (Figure 5C). The CIBERSORT algorithm revealed that the high-risk group with more immune cell infiltrates included T cells CD4 memory activated, Macrophages M2, Dendritic cells activated, and Neutrophils, but low immune cell infiltrates included B cells naive and T cells memory resting (Figure 5D). We then investigated whether the risk signature could predict sensitivity to chemotherapy drugs. Using the CellMiner database, we calculated risk scores for NCI60 cell lines and divided them into high- and low-risk groups delimited by median values. Correlations between risk score and the half maximal inhibitory concentration (IC50) values of FDA-approved drugs were calculated using the Spearman method, which showed that by-products of CUDC-305, Denileukin/Diftitox/Ontak, LDK-378, Nilotinib and Tamoxifen were significantly correlated with the risk score (|cor| > 0.3 and *p*-value <0.05) (Figure 5E). In addition, IC50 values for by-products of CUDC-305, Denileukin/Diftitox/Ontak, and Tamoxifen were lower in the low-risk group (Figure 5F). These findings suggest that the model may be able to act as a chemosensitivity predictor.

### The role of prognostic FR-RBPs in BRCA

**FIGURE 6:**
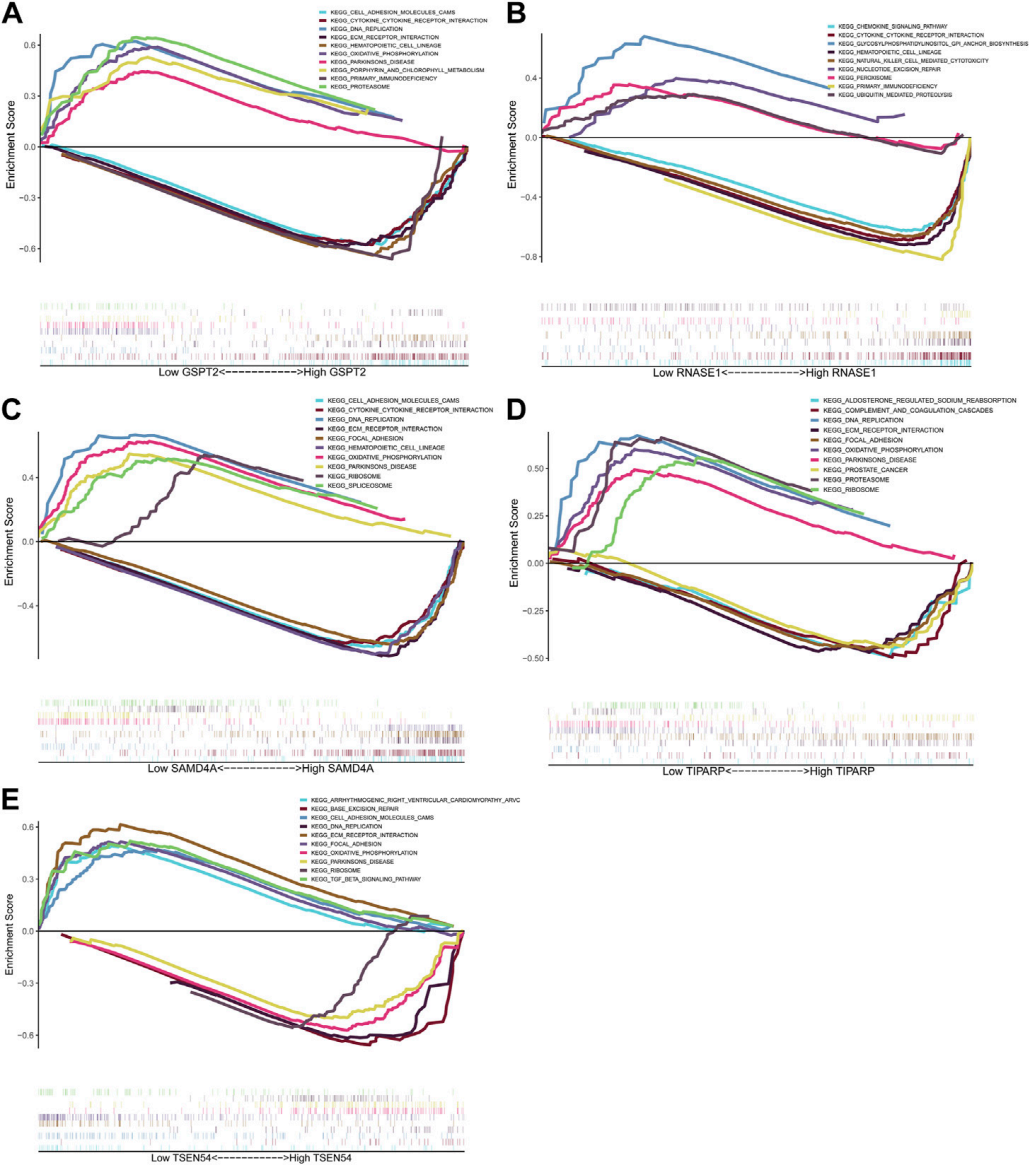
GSEA analysis of prognostic genes. Top5 KEGG pathways significantly enriched in high and low expression groups of GSPT2 **(A)**, RNASE1 **(B)**, SAMD4A **(C)**, TIPARP **(D)** and TSEN54 **(E)**.

We carried out the relevant study by utilizing the cBioPortal database in order to better comprehend the mutations in the five prognostic genes in BRCA. As shown in Supplementary Figure S3, GSPT2, RNASE1, TIPARP, TSEN54, and SAMD4A were mutated in BRCA, with amplification being the predominant mutation type. To further investigate the role of prognostic genes in the BRCA progression, we proceeded with a single-gene GSEA enrichment analysis with detailed information on the results listed in Supplementary Table S9. The top 5 pathways activated in the low expression group and the top 5 pathways activated in the high expression group for each gene are shown in Figures 6A–E. We noted that the low expression groups of GSPT2, SAMD4A, TIPARP, and the high expression group of TSEN54 significantly activated the “oxidative phosphorylation” and ‘DNA replication’ pathways. The high expression group of RNASE1 significantly activated the ‘cytokine receptor interaction’ and ‘natural killer cell mediated cytotoxicity’ pathways. The high expression group of GSPT2, SAMD4A, TIPARP, and the low expression group of TSEN54 significantly activated the “ECM receptor interaction” pathway. The low expression group of TSEN54 significantly activated the “TGF beta signaling pathway”.

### The expression of prognostic FR-RBPs in BRCA

**FIGURE 7:**
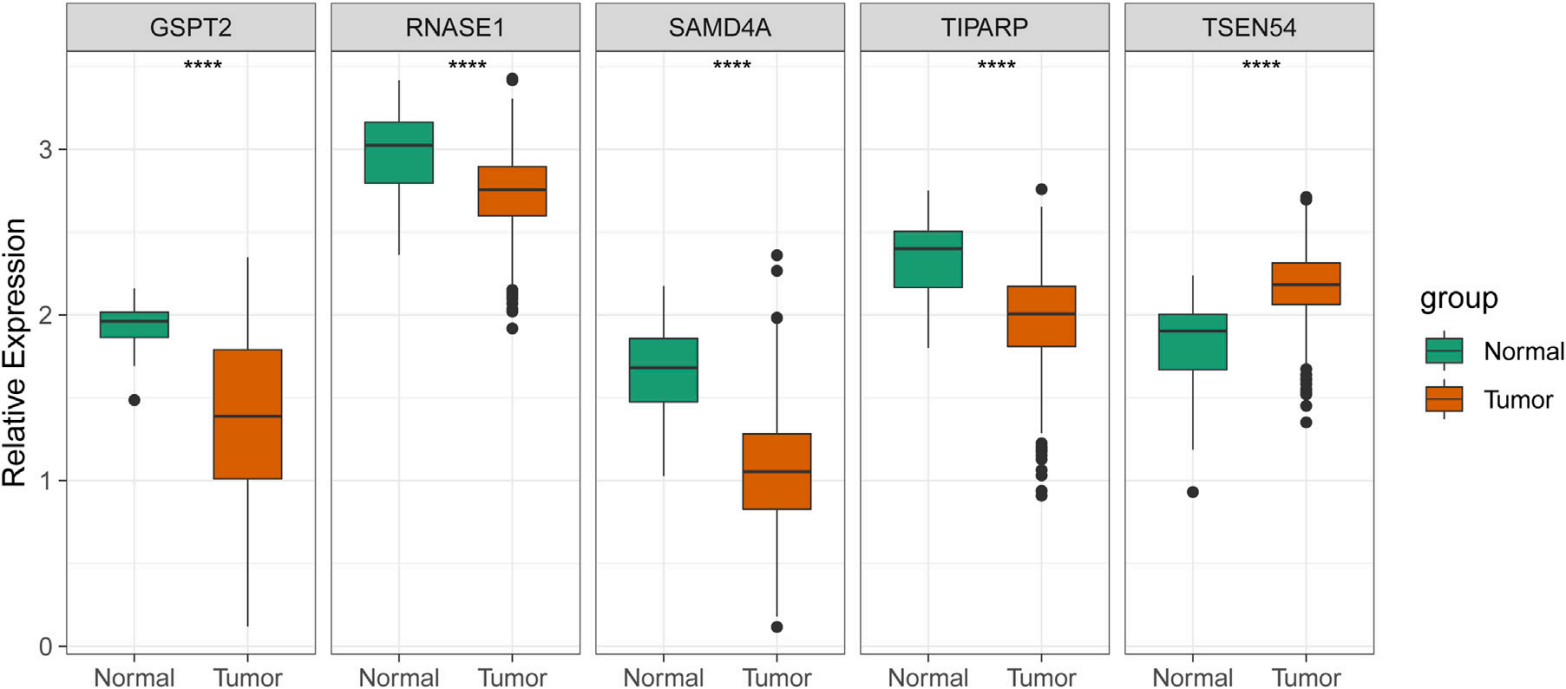
| Validation of the expression of five prognostic genes in the normal and BRCA samples from ICGC cohort. ****p-value < 0.0001.

**FIGURE 8:**
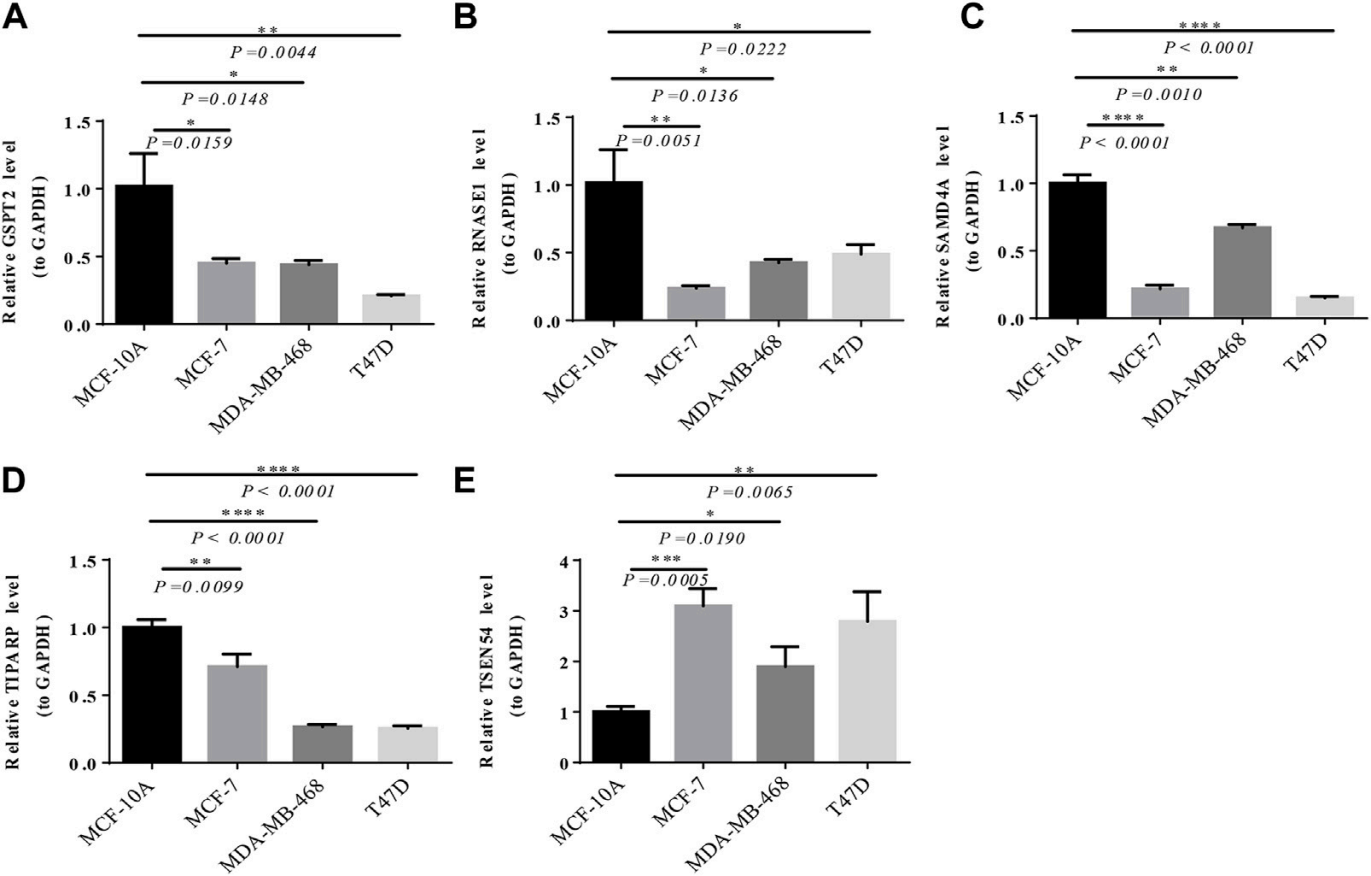
Verification of the expression of prognostic genes by RT-qPCR. The expression of GSPT2 **(A)**, RNASE1 **(B)**, SAMD4A **(C)**, TIPARP **(D)**, and TSEN54 **(E)** among MCF-10A, MCF-7, MDA-MB-468, and T47D. *p-value <0.05, **p-value <0.01, ***p-value <0.001, ****p-value <0.0001.

**FIGURE 9:**
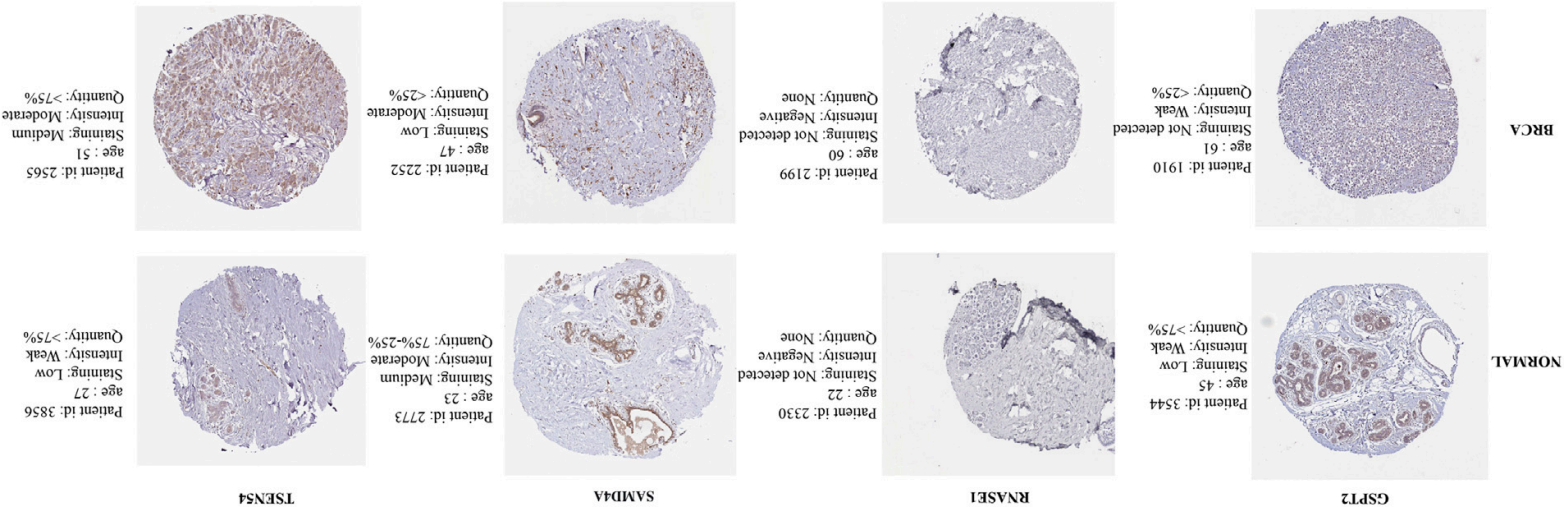
Immunohistochemical results of four prognostic genes in normal and BRCA tissues obtained from HPA database.

As exhibited in Figure 7 **,** TSEN54 was upregulated in BRCA tissues, while GSPT2, RNASE1, TIPARP, and SAMD4A were downregulated in BRCA tissues compared to normal tissues. We then validated the expression of prognostic genes at the mRNA level in human epithelial cell lines from the mammary gland, MCF-10A and three breast cancer cell lines MCF-7, MDA-MB-468, and T47D. Consistent with the trend of results from public databases, TSEN54 was upregulated in breast cancer cell lines and GSPT2, RNASE1, SAMD4A, and TIPARP were downregulated in breast cancer cell lines (Figure 8A-E). To further determine the changes in expression of prognostic genes at the protein level, we obtained corresponding images from the HPA database. We did not detect immunohistochemical results for TIPARP in BRCA. As shown in Figure 9, we found that protein expression levels of GSPT2 and SAMD4A were decreased in BRCA tissues compared to normal tissues, but RNASE1 was largely unexpressed in normal breast tissues and BRCA tissues at protein levels. The increased expression of TSEN54 at protein level in BRCA tissues compared to normal tissues was noteworthy.

### The survival status of signature genes

**FIGURE 10:**
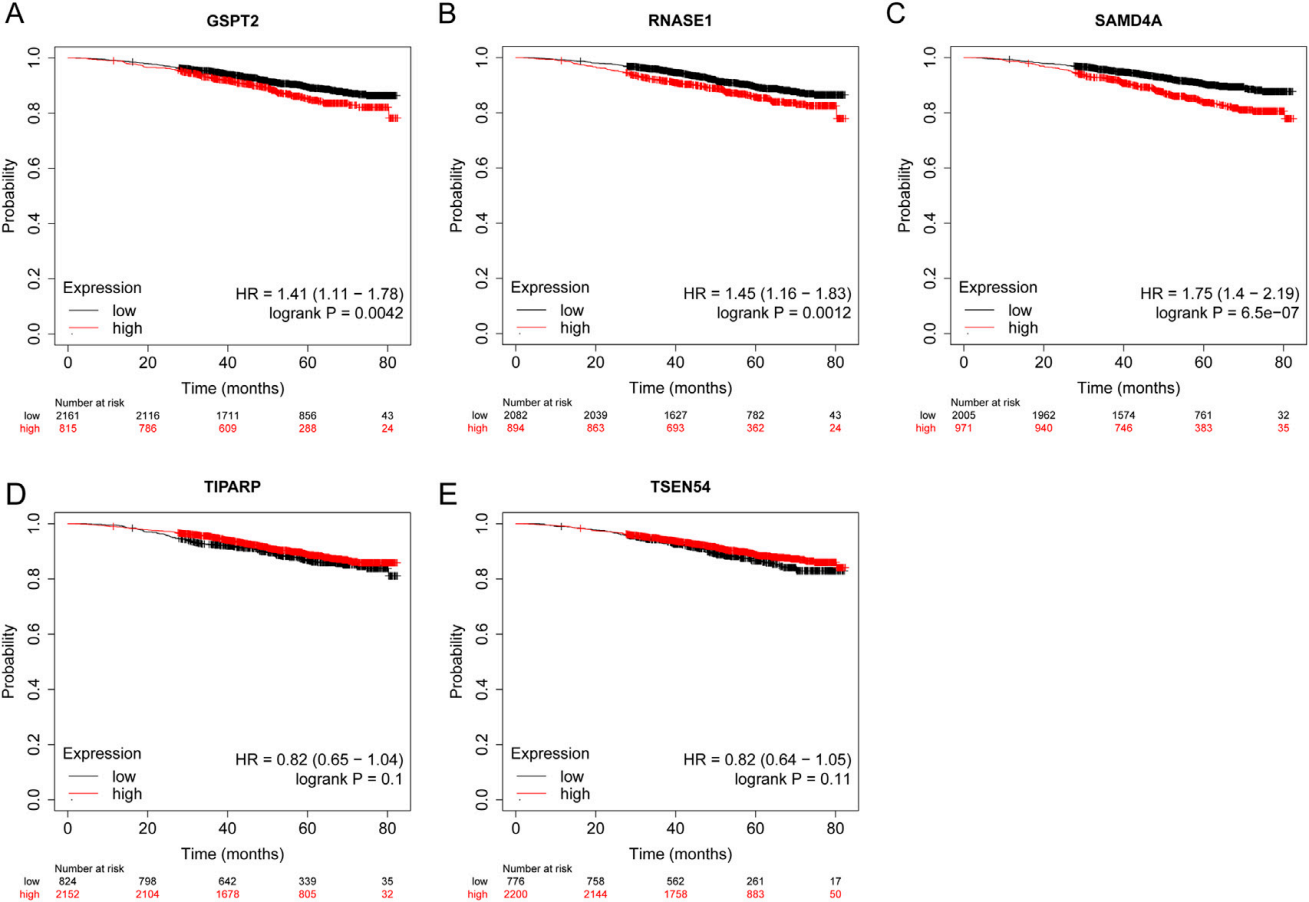
The prognostic value of five model genes in BRCA Patients using the Kaplan-Meier plotter database. The survival probability of patients in high- and low-expression groups divided by the transcriptional expression levels of GSPT2 **(A)**, RNASE1 **(B)**, SAMD4A **(C)**, TIPARP **(D)**, and **(E)** TSEN54. The red colored lines indicate high expression levels while the black colored lines indicate low expression levels.

KM plotter database was utilized to analyze the OS of BRCA patients. The log-rank test and KM curve analyses suggested that the high expression level of GSPT2 (HR = 1.41, *p* = 0.0042), RNASE1 (HR = 1.45, *p* = 0.0012), and SAMD4A (HR = 1.75, *p* < 0.001) were significant with the poor OS of the BRCA patients. While the expression levels of TIPARP and TSEN54 were not associated with OS of BRCA patients (Figure 10A-E).

## Discussion

BRCA is the most common factor in cancer deaths in women globally. Even though the prognosis for BRCA patients has significantly improved due to advancements in numerous treatment modalities such as local surgery, radiation, chemotherapy, and endocrine therapy, the risk of recurrence and death still exists for many individuals (Dong et al., 2018; Wang et al., 2020a). In clinical settings, the prognosis of BRCA patients is frequently predicted using the tumor size, tumor grade, and TNM stage (Al-Keilani et al., 2021). However, because of individual variability, these clinical markers frequently fall short of the ideal prediction effect and the overall diagnostic standards. In recent years, many researchers have been exploring new biomarkers that can predict the prognosis of BRCA at the molecular level, and we have made new discoveries in this area.

Functional enrichment analysis was used to determine the biological roles of these DE-FR-RBPs. Little is known about the specific mechanism of action of the spliceosome in cancer. In the past, it was found that there is a high frequency of mutations in different components of the spliceosome in chronic granulocytic leukemia (Quesada et al., 2012), and deregulation of RNA splicing is common in many tumor transcriptomes (Dvinge and Bradley, 2015; Kahles et al., 2018), suggesting the development of cancer may be influenced by this biological process (Ebert and Bernard, 2011). Similarly, a recent study in BRCA showed that mutations in key genes in the spliceosome lead to a BRCA-like cell phenotype (Lappin et al., 2022). In our study, upregulated genes were significantly enriched for biological processes associated with spliceosomes. Nucleases are key components of biological processes and are expressed at both gene and protein levels in cancer cells (Doherty and Madhusudan, 2015; He et al., 2016), and the failure of nuclease activity can lead to genomic instability and susceptibility to many cancers, including BRCA (Zheng et al., 2007). The mRNA metabolic pathways include mRNA transport, pre-mRNA splicing, RNA editing, mRNA degradation, and translation activation (Zou et al., 2018). Numerous regulatory proteins are involved in these biological processes (Niu et al., 2020), and some of these have been demonstrated to have intricate roles in cancer; for example, m6A regulatory proteins can induce oncogene expression, cancer cell proliferation, survival, and tumorigenesis and development (Sun et al., 2019; Wang et al., 2020b). Translation regulation is a critical stage in the development and progression of cancer, which mediates the overall expression of protein synthesis as well as the precise translation of certain mRNAs that may promote a variety of oncogenic characteristics, such as cell transformation, tumor cell survival, invasion, metastasis, and angiogenesis (Khan et al., 2016). In conclusion, this is consistent with our findings that various genes, chemicals, and pathways are involved in the development of BRCA.

The prognostic model proposed in this study consists of five DE-FR-RBPs (TIPARP, SAMD4A, TSEN54, GSPT2, and RNASE1). Several studies have demonstrated the significant role that these genes play in the development of numerous malignancies, including BRCA. TIPARP acts as a tumor inhibitor by down-regulating pro-tumor transcription factors. Therefore, small molecules that increase the expression or activity of TIPARP may be effective anticancer drugs (Zhang et al., 2020a). In addition, the expression of TIPARP is negatively correlated with the methylation status, and DNA methylation may be an important mechanism for the dysregulation of TIPARP in BRCA. It is worth noting that the expression of TIPARP is significantly reduced in BRCA, and more advanced BRCA tends to express lower levels of TIPARP, compared with adjacent normal tissues. The expression of TIPARP in BRCA tissues has always been low (Lin Cheng et al., 2019). SAMD4A is a novel breast tumor suppressor, significantly inhibiting breast tumor-induced angiogenesis by disrupting the balance of angiogenesis-related genes in tumor cells (Zhou et al., 2021). TSEN54 is involved in the complex process of pre-tRNA splicing site identification and cutting and may be related to the survival rate of BRCA patients (Lou et al., 2021; Yan et al., 2022). Further research is required to fully understand its mechanism of action in BRCA. We originally reported GSPT2 as a protective factor linked with BRCA based on current prognostic models, as there is no research on the role of GSPT2 in BRCA. In comparison to liver cancer patients, the normal control group’s blood had greater levels of GSPT2 expression in other malignancies (Li et al., 2014). Similarly, GSPT2 was discovered to be the most distinct and singular factor in stage II colorectal cancer patients when evaluating the difference in gene expression between stage II and stage III patients (Groene et al., 2006). RNASE1 can regulate the vascular homeostasis of extracellular RNA (Bedenbender et al., 2019). In prostate cancer, the overexpression of RNASE1 is associated with poor survival (Gao et al., 2020). Li et al. pointed out that RNASE1 was highly expressed in BRCA (Li et al., 2020b). However, further research is needed to confirm its specific mechanism of action in BRCA.

TMB is defined as the number of mutations per DNA giant base (Greillier et al., 2018). In this study, we found that the TMB, related to immunotherapy, in the high-risk group was significantly higher than that in the low-risk group. We found that NRP1, CD244, ADORA2A, TNFRSF14, and TNFRSF15 were sparsely expressed in the high-risk group by comparing the expression of immune checkpoint molecules in the high-risk and low-risk groups. LAG3 was highly expressed in the high-risk group. NRP1 is a potential molecule that can modify the tumor microenvironment (TME) and innate immune system in a targeted way to produce an efficient anti-tumor immune response, which binds to and uptakes neutrophil elastin on a number of BRCA cell lines (Kerros et al., 2017). One of the immune cell receptors that CD244 regulates is NK cell toxicity (Buller et al., 2020). Expression of CD244 is linked to the generation of inhibitory molecules and the inhibition of antigen-specific CD8+T cell activity, primarily spreading inhibitory signals in immune cells linked with tumors and immunosuppression in tumor microenvironment experiences (Agresta et al., 2018). ADORA2A, an immunological checkpoint molecule, has been found to have a high association with the majority of female cancers, including BRCA (Wan et al., 2022). Adenosine mediates TME immunosuppression of immune cells *via* ADORA2A, and in addition, drugs targeting ADORA2A have entered phase I clinical trials for immunotherapy of renal cell carcinoma (Fong et al., 2020; Feng et al., 2022). In the tumor microenvironment, TNFRSF14 is crucial for immune system activation and recruitment (Lombardo et al., 2020). Studies of diffuse large B-cell lymphomas have revealed that TNFRSF14 changes are described as early driver mutations and accelerated mutations that may occur in the early stages of the disease, considerably earlier than the diagnosis of malignant lymphoma (Vogelsberg et al., 2020). This further strengthens our observations and suggests that similar mechanisms can be shared in BRCA. LAG3 builds up in many malignancies, and the activity of this protein is associated with immune cell infiltration, proliferation, and secretion (Shi et al., 2022). A favorable prognosis is connected with cutting the surface portion of LAG3, which weakens the lymphocytes’ inhibitory signal (Du et al., 2020). The immunological checkpoint molecule LAG3 was also shown to be substantially expressed in breast tumor tissues as compared to the normal control group, according to BRCA research (Sasidharan Nair et al., 2018), which is consistent with what we discovered.

Clinically, susceptibility to chemotherapy drugs differs from person to person; even BRCA patients with the same stage of cancer will react differentially to the same treatment regimen. Breast tumors are often categorized using lymph node metastasis and histological grade in order to apply various treatment regimens. However, this approach lacks accuracy, as 70%–80% of patients survive without these treatments (van’t Veer et al., 2002). Therefore, the finest individualized treatment programs for diverse people before chemotherapy will have an impact on the patient’s prognosis. In comparison to a single gene alone, many gene combinations may predict chemotherapy sensitivity with higher sensitivity and specificity (Zhang et al., 2020b). In this study, our risk model based on TIPARP, SAMD4A, TSEN54, GSPT2, and RNASE1 was compared with the high and low risk groups of breast cancer cell lines in the CellMiner database. IC50 values for by-products of CUDC-305, Denileukin/Diftitox/Ontak, and Tamoxifen were lower in the low-risk group. A lower IC50, in our opinion, implies that these medications function more efficiently. Our study may enable physicians to customize medicine for each patient by assisting them in predicting the therapeutic response of BRCA patients before they undergo treatment.

Previous studies have shown that gene mutations are the causes of cancer (Frixa et al., 2015; Liu et al., 2017), with gene amplification being the most common genetic change associated with cancer (Wu et al., 2020). According to our analysis of the cBioPortal database, TIPARP, SAMD4A, TSEN54, GSPT2, and RNASE1 are all mutated in BRCA, and gene amplification is the main mutation type. This study’s GSEA enrichment analysis leads to the conclusion that certain biological processes are crucial to the development of cancer. In many malignant tumors, loss of tumor suppressor factors and inactivation of some proto-oncogenes can induce oxidative phosphorylation (Becherini et al., 2021). In BRCA, breast cancer stem cells upregulate oxidative phosphorylation signals and rely on oxidative phosphorylation phenotypes to meet their own high metabolic requirements. Therefore, biological processes that inhibit oxidative phosphorylation can produce tumor suppressive effects (Raninga et al., 2020). DNA replication is the basis of all tissues, including tumor tissue. A study of BRCA has shown that cancer cells somehow improve or compensate for any potential DNA replication and/or repair defects (Lee et al., 1999). The cytokine-cytokine receptor interaction is a key pathway for regulating cellular inflammation (He et al., 2019). Many studies have supported the role of inflammatory response in BRCA (Guaita-Esteruelas et al., 2017; Pham et al., 2020; Zhang et al., 2021). Natural killer cells are lymphocytes of the natural immune system that have cytotoxic activity and help eliminate pathogen-induced infections and cancer cells (Al Absi et al., 2018). The natural killer cell-mediated cytotoxicity pathway is associated with NK cell activation, which inhibits tumor development and eradicates malignancies (Yoon et al., 2015; Paul and Lal, 2017). Tumor adhesion, abscission, disintegration, motility, and proliferation are all impacted by the ECM receptor interaction pathway (Bao et al., 2019). ECM plays a role in gastric cancer invasion and metastasis as well as the promotion of epithelial-mesenchymal transition in colorectal cancer cells (Rahbari et al., 2016; Yan et al., 2018). In breast tumor tissues, ECM proteins or genes are significantly expressed (Yoon et al., 2015). TGF-β is a key regulator of epithelial-to-mesenchymal transformation, and TGF-β signal also upregulates a series of oncogenic genes to further promote metastasis. Therefore, inhibition of TGF-β signal transduction is conducive to inhibiting BRCA metastasis (Tang et al., 2017). In addition, among the prognostic genes screened in this study, GSPT2, RNASE1, and SAMD4A are downregulated in the Cytokine-Receptor Interaction pathway, and cytokines play a broad range of immunomodulatory roles critical to human biology and disease (Spangler et al., 2015). GSPT2, SAMD4A, and TIPARP are downregulated in ECM-receptor interaction pathways, which play important roles in tumor shedding, adhesion, degradation, motility, and proliferation and may be involved in breast cancer development (Bao et al., 2019). High expression levels of ECM proteins or genes in breast tumor tissues may provide new ideas for cancer therapy. We believe that these genes and pathways may be potential markers of breast cancer, but the mechanisms of tumorigenesis and progression need further experimental validation.

In addition, we acknowledge limitations in our work. First, because our data were obtained from public sources, certain clinical details, such as patient treatment information, were omitted to allow for more detailed analyses of the data for comparison purposes. Second, clinical application studies and more experimental mechanism studies are lacking. We will continue to focus on these topics in the next studies as well.

In summary, we identified key genes and related pathways through bioinformatics analysis of differential expression of DE-FR-RBPs in BRCA. We established a new prediction model based on the key genes. ROC analysis further proved that this method was reliable in predicting the prognosis of BRCA patients. Furthermore, we found that the FR-RBPs-associated risk signature was an independent and effective predictor of BRCA survival. Subsequently, we verified the consistency of transcription levels in clinical samples and explored trends in the expression of prognostic genes at the protein level in BRCA and normal tissues. These results may make it easier to understand the mechanisms of BRCA and help us explore new biomarkers for BRCA patients.

## Data Availability

The original contributions presented in the study are included in the article/Supplementary Material, further inquiries can be directed to the corresponding authors.

## References

[B1] Agresta L. Hoebe K. H. N. Janssen E. M. (2018). The emerging role of CD244 signaling in immune cells of the tumor microenvironment. Front. Immunol. 9, 2809. 10.3389/fimmu.2018.02809 30546369 PMC6279924

[B2] Al Absi A. Wurzer H. Guerin C. Hoffmann C. Moreau F. Mao X. (2018). Actin cytoskeleton remodeling drives breast cancer cell escape from natural killer–mediated cytotoxicity. Cancer Res. 78, 5631–5643. 10.1158/0008-5472.CAN-18-0441 30104240

[B3] Al-Keilani M. S. Elstaty R. I. Alqudah M. A. Alkhateeb A. M. (2021). Immunohistochemical expression of substance P in breast cancer and its association with prognostic parameters and Ki-67 index. PLoS ONE 16, e0252616. 10.1371/journal.pone.0252616 34086748 PMC8177477

[B4] Bao Y. Wang L. Shi L. Yun F. Liu X. Chen Y. (2019). Transcriptome profiling revealed multiple genes and ECM-receptor interaction pathways that may be associated with breast cancer. Cell Mol. Biol. Lett. 24, 38. 10.1186/s11658-019-0162-0 31182966 PMC6554968

[B5] Becherini P. Caffa I. Piacente F. Damonte P. Vellone V. G. Passalacqua M. (2021). SIRT6 enhances oxidative phosphorylation in breast cancer and promotes mammary tumorigenesis in mice. Cancer Metab. 9, 6. 10.1186/s40170-021-00240-1 33482921 PMC7821730

[B6] Bedenbender K. Scheller N. Fischer S. Leiting S. Preissner K. T. Schmeck B. T. (2019). Inflammation‐mediated deacetylation of the ribonuclease 1 promoter *via* histone deacetylase 2 in endothelial cells. FASEB J. 33, 9017–9029. 10.1096/fj.201900451R 31039328

[B7] Buller C. W. Mathew P. A. Mathew S. O. (2020). Roles of NK cell receptors 2B4 (CD244), CS1 (CD319), and LLT1 (CLEC2D) in cancer. Cancers 12, 1755. 10.3390/cancers12071755 32630303 PMC7409338

[B8] Doherty R. Madhusudan S. (2015). DNA repair endonucleases: Physiological roles and potential as drug targets. SLAS Discov. 20, 829–841. 10.1177/1087057115581581 25877151

[B9] Dong H. Wang W. Chen R. Zhang Y. Zou K. Ye M. (2018). Exosome-mediated transfer of lncRNA-SNHG14 promotes trastuzumab chemoresistance in breast cancer. Int. J. Oncol. 53, 1013–1026. 10.3892/ijo.2018.4467 30015837 PMC6065402

[B10] Du F. Qiao C. Li X. Chen Z. Liu H. Wu S. (2019). Forkhead box K2 promotes human colorectal cancer metastasis by upregulating ZEB1 and EGFR. Theranostics 9, 3879–3902. 10.7150/thno.31716 31281520 PMC6587343

[B11] Du H. Yi Z. Wang L. Li Z. Niu B. Ren G. (2020). The co-expression characteristics of LAG3 and PD-1 on the T cells of patients with breast cancer reveal a new therapeutic strategy. Int. Immunopharmacol. 78, 106113. 10.1016/j.intimp.2019.106113 31841754

[B12] Dvinge H. Bradley R. K. (2015). Widespread intron retention diversifies most cancer transcriptomes. Genome Med. 7, 45. 10.1186/s13073-015-0168-9 26113877 PMC4480902

[B13] Early Breast Cancer Trialists’ Collaborative Group (EBCTCG) (2011). Relevance of breast cancer hormone receptors and other factors to the efficacy of adjuvant tamoxifen: Patient-level meta-analysis of randomised trials. Lancet 378, 771–784. 10.1016/s0140-6736(11)60993-8 21802721 PMC3163848

[B14] Ebert B. Bernard O. A. (2011). Mutations in RNA splicing machinery in human cancers. N. Engl. J. Med. 365, 2534–2535. 10.1056/NEJMe1111584 22150007

[B15] Feng D. Shi X. Zhang F. Xiong Q. Wei Q. Yang L. (2022). Energy metabolism-related gene prognostic index predicts biochemical recurrence for patients with prostate cancer undergoing radical prostatectomy. Front. Immunol. 13, 839362. 10.3389/fimmu.2022.839362 35280985 PMC8908254

[B16] Fong L. Hotson A. Powderly J. D. Sznol M. Heist R. S. Choueiri T. K. (2020). Adenosine 2A receptor blockade as an immunotherapy for treatment-refractory renal cell cancer. Cancer Discov. 10, 40–53. 10.1158/2159-8290.CD-19-0980 31732494 PMC6954326

[B17] Frixa T. Donzelli S. Blandino G. (2015). Oncogenic MicroRNAs: Key players in malignant transformation. Cancers 7, 2466–2485. 10.3390/cancers7040904 26694467 PMC4695904

[B18] Gao L. Meng J. Zhang Y. Gu J. Han Z. Wang X. (2020). Development and validation of a six-RNA binding proteins prognostic signature and candidate drugs for prostate cancer. Genomics 112, 4980–4992. 10.1016/j.ygeno.2020.08.034 32882325

[B19] Greillier L. Tomasini P. Barlesi F. (2018). The clinical utility of tumor mutational burden in non-small cell lung cancer. Transl. Lung Cancer Res. 7, 639–646. 10.21037/tlcr.2018.10.08 30505708 PMC6249623

[B20] Groene J. Mansmann U. Meister R. Staub E. Roepcke S. Heinze M. (2006). Transcriptional census of 36 microdissected colorectal cancers yields a gene signature to distinguish UICC II and III. Int. J. Cancer 119, 1829–1836. 10.1002/ijc.22027 16721809

[B21] Guaita-Esteruelas S. Saavedra-García P. Bosquet A. Borras J. Girona J. Amiliano K. (2017). Adipose-derived fatty acid-binding proteins plasma concentrations are increased in breast cancer patients. Oncol. 22, 1309–1315. 10.1634/theoncologist.2016-0483 PMC5679823 28701570

[B22] He L. Zhang Y. Sun H. Jiang F. Yang H. Wu H. (2016). Targeting DNA flap endonuclease 1 to impede breast cancer progression. EBioMedicine 14, 32–43. 10.1016/j.ebiom.2016.11.012 27852524 PMC5161424

[B23] He Y. Shi J. Nguyen Q. T. You E. Liu H. Ren X. (2019). Development of highly potent glucocorticoids for steroid-resistant severe asthma. Proc. Natl. Acad. Sci. U. S. A. 116, 6932–6937. 10.1073/pnas.1816734116 30894497 PMC6452690

[B24] Jin Y. Zhang Y. Li B. Zhang J. Dong Z. Hu X. (2019). TRIM21 mediates ubiquitination of Snail and modulates epithelial to mesenchymal transition in breast cancer cells. Int. J. Biol. Macromol. 124, 846–853. 10.1016/j.ijbiomac.2018.11.269 30502437

[B25] Kahles A. Lehmann K-V. Toussaint N. C. Huser M. Stark S. G. Sachsenberg T. (2018). Comprehensive analysis of alternative splicing across tumors from 8,705 patients. Cancer Cell 34, 211–224.e6. 10.1016/j.ccell.2018.07.001 30078747 PMC9844097

[B26] Kerros C. Tripathi S. C. Zha D. Mehrens J. M. Sergeeva A. Philips A. V. (2017). Neuropilin-1 mediates neutrophil elastase uptake and cross-presentation in breast cancer cells. J. Biol. Chem. 292, 10295–10305. 10.1074/jbc.M116.773051 28468826 PMC5473232

[B27] Khan S. Imran A. Khan A. A. Abul Kalam M. Alshamsan A. (2016). Systems biology approaches for the prediction of possible role of Chlamydia pneumoniae proteins in the etiology of lung cancer. PLoS ONE 11, e0148530. 10.1371/journal.pone.0148530 26871581 PMC4752481

[B28] Lan Y. Su J. Xue Y. Zeng L. Cheng X. Zeng L. (2021). Analysing a novel RNA-binding-protein-related prognostic signature highly expressed in breast cancer. J. Healthc. Eng. 2021, 9174055–9714070. 10.1155/2021/9174055 34707800 PMC8545572

[B29] Lappin K. M. Barros E. M. Jhujh S. S. Irwin G. W. McMillan H. Liberante F. G. (2022). Cancer-associated SF3B1 mutations confer a BRCA-like cellular phenotype and synthetic lethality to PARP inhibitors. Cancer Res. 82, 819–830. 10.1158/0008-5472.CAN-21-1843 35027467 PMC7612475

[B30] Lee H. Trainer A. H. Friedman L. S. Thistlethwaite F. C. Evans M. J. Ponder B. A. (1999). Mitotic checkpoint inactivation fosters transformation in cells lacking the breast cancer susceptibility gene, Brca2. Mol. Cell 4, 1–10. 10.1016/s1097-2765(00)80182-3 10445022

[B31] Li M. Wang J. Yang L. Gao P. Tian Q. B. Liu D. W. (2014). eRF3b, a biomarker for hepatocellular carcinoma, influences cell cycle and phosphoralation status of 4E-BP1. PLoS ONE 9, e86371. 10.1371/journal.pone.0086371 24466059 PMC3900531

[B32] Li P. Yang B. Xiu B. Chi Y. Xue J. Wu J. (2021). Development and validation of a robust ferroptosis-related gene panel for breast cancer disease-specific survival. Front. Cell Dev. Biol. 9, 709180. 10.3389/fcell.2021.709180 34900981 PMC8655913

[B33] Li S. Jiang L. Tang J. Gao N. Guo F. (2020). Kernel fusion method for detecting cancer subtypes via selecting relevant expression data. Front. Genet. 11, 979. 10.3389/fgene.2020.00979 33133130 PMC7511763

[B34] Li W. Gao L-N. Song P-P. You C. G. (2020). Development and validation of a RNA binding protein-associated prognostic model for lung adenocarcinoma. Aging 12, 3558–3573. 10.18632/aging.102828 32087603 PMC7066909

[B35] Lin Cheng L. Li Z. Huang Y-Z. Zhang X. Dai X. Y. Shi L. (2019). TCDD-inducible poly-ADP-ribose polymerase (TIPARP), A novel therapeutic target of breast cancer. CMAR 11, 8991–9004. 10.2147/CMAR.S219289 PMC6805248 31695491

[B36] Liu H. Zhou Y. Liu Q. Xiao G. Wang B. Li W. (2017). Association of miR-608 rs4919510 polymorphism and cancer risk: A meta-analysis based on 13,664 subjects. Oncotarget 8, 37023–37031. 10.18632/oncotarget.9509 27223084 PMC5514889

[B37] Liu Q. Ma J-Y. Wu G. (2021). Identification and validation of a ferroptosis-related gene signature predictive of prognosis in breast cancer. Aging 13, 21385–21399. 10.18632/aging.203472 34499616 PMC8457571

[B38] Livak K. J. Schmittgen T. D. (2001). Analysis of relative gene expression data using real-time quantitative PCR and the 2(-Delta Delta C(T)) Method. Methods 25, 402–408. 10.1006/meth.2001.1262 11846609

[B39] Lombardo S. Bramanti A. Ciurleo R. Basile M. S. Pennisi M. Bella R. (2020). Profiling of inhibitory immune checkpoints in glioblastoma: Potential pathogenetic players. Oncol. Lett. 20, 332. 10.3892/ol.2020.12195 33123243 PMC7583708

[B40] Lou S. Meng F. Yin X. Zhang Y. Han B. Xue Y. (2021). Comprehensive characterization of RNA processing factors in gastric cancer identifies a prognostic signature for predicting clinical outcomes and therapeutic responses. Front. Immunol. 12, 719628. 10.3389/fimmu.2021.719628 34413861 PMC8369824

[B41] Luo H. Zhang Y. Hu N. He Y. He C. (2021). Systematic construction and validation of an RNA-binding protein-associated prognostic model for acute myeloid leukemia. Front. Genet. 12, 715840. 10.3389/fgene.2021.715840 34630514 PMC8498117

[B42] Niu X. Xu J. Liu J. Chen L. Qiao X. Zhong M. (2020). Landscape of N6-methyladenosine modification patterns in human ameloblastoma. Front. Oncol. 10, 556497. 10.3389/fonc.2020.556497 33178585 PMC7592903

[B43] Paul S. Lal G. (2017). The molecular mechanism of natural killer cells function and its importance in cancer immunotherapy. Front. Immunol. 8, 1124. 10.3389/fimmu.2017.01124 28955340 PMC5601256

[B44] Peng Y. Yu H. Zhang Y. Qu F. Tang Z. Qu C. (2021). A ferroptosis-associated gene signature for the prediction of prognosis and therapeutic response in luminal-type breast carcinoma. Sci. Rep. 11, 17610. 10.1038/s41598-021-97102-z 34475496 PMC8413464

[B45] Pham D-V. Raut P. K. Pandit M. Chang J. H. Katila N. Choi D. Y. (2020). Globular adiponectin inhibits breast cancer cell growth through modulation of inflammasome activation: Critical role of Sestrin2 and AMPK signaling. Cancers 12, 613. 10.3390/cancers12030613 32155890 PMC7139717

[B46] Quesada V. Conde L. Villamor N. Ordonez G. R. Jares P. Bassaganyas L. (2012). Exome sequencing identifies recurrent mutations of the splicing factor SF3B1 gene in chronic lymphocytic leukemia. Nat. Genet. 44, 47–52. 10.1038/ng.1032 22158541

[B47] Rahbari N. N. Kedrin D. Incio J. Liu H. Ho W. W. Nia H. T. (2016). Anti-VEGF therapy induces ECM remodeling and mechanical barriers to therapy in colorectal cancer liver metastases. Sci. Transl. Med. 8, 360ra135. 10.1126/scitranslmed.aaf5219 PMC5457741 27733559

[B48] Raninga P. V. Lee A. Sinha D. Dong L. F. Datta K. K. Lu X. (2020). Marizomib suppresses triple-negative breast cancer via proteasome and oxidative phosphorylation inhibition. Theranostics 10, 5259–5275. 10.7150/thno.42705 32373211 PMC7196287

[B49] Ritchie M. E. Phipson B. Wu D. Hu Y. Law C. W. Shi W. (2015). Limma powers differential expression analyses for RNA-sequencing and microarray studies. Nucleic Acids Res. 43, e47. 10.1093/nar/gkv007 25605792 PMC4402510

[B50] Sasidharan Nair V. El Salhat H. Taha R. Z. John A. Ali B. R. Elkord E. (2018). DNA methylation and repressive H3K9 and H3K27 trimethylation in the promoter regions of PD-1, CTLA-4, TIM-3, LAG-3, TIGIT, and PD-L1 genes in human primary breast cancer. Clin. Epigenet 10, 78. 10.1186/s13148-018-0512-1 PMC6003083 29983831

[B51] Shi A-P. Tang X-Y. Xiong Y-L. Zheng K. F. Liu Y. J. Shi X. G. (2022). Immune checkpoint LAG3 and its ligand FGL1 in cancer. Front. Immunol. 12, 785091. 10.3389/fimmu.2021.785091 35111155 PMC8801495

[B52] Siegel R. L. Miller K. D. Jemal A. (2020). Cancer statistics, 2020. CA A Cancer J. Clin. 70, 7–30. 10.3322/caac.21590 31912902

[B53] Song J. Liu Y. Guan X. Zhang X. Yu W. Li Q. (2021). A novel ferroptosis-related biomarker signature to predict overall survival of esophageal squamous cell carcinoma. Front. Mol. Biosci. 8, 675193. 10.3389/fmolb.2021.675193 34291083 PMC8287967

[B54] Spangler J. B. Moraga I. Mendoza J. L. Garcia K. C. (2015). Insights into cytokine–receptor interactions from cytokine engineering. Annu. Rev. Immunol. 33, 139–167. 10.1146/annurev-immunol-032713-120211 25493332 PMC4445396

[B55] Sun T. Wu R. Ming L. (2019). The role of m6A RNA methylation in cancer. Biomed. Pharmacother. 112, 108613. 10.1016/j.biopha.2019.108613 30784918

[B56] Sung H. Ferlay J. Siegel R. L. Laversanne M. Soerjomataram I. Jemal A. (2021). Global cancer statistics 2020: GLOBOCAN estimates of incidence and mortality worldwide for 36 cancers in 185 countries. CA A Cancer J. Clin. 71, 209–249. 10.3322/caac.21660 33538338

[B57] Tang X. Shi L. Xie N. Liu Z. Qian M. Meng F. (2017). SIRT7 antagonizes TGF-β signaling and inhibits breast cancer metastasis. Nat. Commun. 8, 318. 10.1038/s41467-017-00396-9 28827661 PMC5566498

[B58] Tian B. Hou M. Zhou K. Qiu X. Du Y. Gu Y. (2021). A novel TCGA-validated, MiRNA-based signature for prediction of breast cancer prognosis and survival. Front. Cell Dev. Biol. 9, 717462. 10.3389/fcell.2021.717462 34589485 PMC8473752

[B59] van't Veer L. J. Dai H. van de Vijver M. J. He Y. D. Hart A. A. M. Mao M. (2002). Gene expression profiling predicts clinical outcome of breast cancer. Nature 6871, 530–536. 10.1038/415530a 11823860

[B60] Vogelsberg A. Steinhilber J. Mankel B. Federmann B. Schmidt J. Montes-Mojarro I. A. (2020). Genetic evolution of *in situ* follicular neoplasia to aggressive B-cell lymphoma of germinal center subtype. haematol 106, 2673–2681. 10.3324/haematol.2020.254854 PMC8485666 32855278

[B61] Waks A. G. Winer E. P. (2019). Breast cancer treatment: A review. JAMA 321, 288–300. 10.1001/jama.2018.19323 30667505

[B62] Wan F. Chen F. Fan Y. Chen D. (2022). Clinical significance of TET2 in female cancers. Front. Bioeng. Biotechnol. 10, 790605. 10.3389/fbioe.2022.790605 35223782 PMC8874273

[B63] Wang L. Chen S. Shen X. Li D. C. Liu H. Y. Ji Y. L. (2020). M6A RNA methylation regulator HNRNPC contributes to tumorigenesis and predicts prognosis in glioblastoma multiforme. Front. Oncol. 10, 536875. 10.3389/fonc.2020.536875 33134160 PMC7578363

[B64] Wang Q. Zhao S. Gan L. Zhuang Z. (2020). Bioinformatics analysis of prognostic value of *PITX1* gene in breast cancer. Biosci. Rep. 40, BSR20202537. 10.1042/BSR20202537 32830857 PMC7494990

[B65] Wang W. Xu S. Zhu X. Guo Q. Y. Zhu M. Mao X. L. (2021). Identification and validation of a novel RNA-binding protein-related gene-based prognostic model for multiple myeloma. Front. Genet. 12, 665173. 10.3389/fgene.2021.665173 33981333 PMC8107400

[B66] Wu S. Li G. Zhao X. Xiang J. Lizaso A. Ye J. (2020). High-level gain of mesenchymal-epithelial transition factor (MET) copy number using next-generation sequencing as a predictive biomarker for MET inhibitor efficacy. Ann. Transl. Med. 8, 685. 10.21037/atm-20-2741 32617305 PMC7327325

[B67] Yan P. He Y. Xie K. Kong S. Zhao W. (2018). *In silico* analyses for potential key genes associated with gastric cancer. PeerJ 6, e6092. 10.7717/peerj.6092 30568862 PMC6287586

[B68] Yan X. You S-N. Chen Y. Qian K. (2022). Construction and validation of a newly prognostic signature for CRISPR-cas9-based cancer dependency map genes in breast cancer. J. Oncol. 2022, 4566577–4566593. 10.1155/2022/4566577 35096059 PMC8791742

[B69] Yoon S. R. Kim T-D. Choi I. (2015). Understanding of molecular mechanisms in natural killer cell therapy. Exp. Mol. Med. 47, e141. 10.1038/emm.2014.114 25676064 PMC4346487

[B70] Yu G. Wang L-G. Han Y. He Q. Y. (2012). clusterProfiler: an R Package for comparing biological themes among gene clusters. OMICS A J. Integr. Biol. 16, 284–287. 10.1089/omi.2011.0118 PMC3339379 22455463

[B71] Zhang L. Cao J. Dong L. Lin H. (2020). TiPARP forms nuclear condensates to degrade HIF-1α and suppress tumorigenesis. Proc. Natl. Acad. Sci. U. S. A. 117, 13447–13456. 10.1073/pnas.1921815117 32482854 PMC7306777

[B72] Zhang Q. Gao C. Shao J. Wang Z. (2021). TIGIT-related transcriptome profile and its association with tumor immune microenvironment in breast cancer. Biosci. Rep. 41, BSR20204340. 10.1042/BSR20204340 33721026 PMC7990089

[B73] Zhang Y. Yuan Z. Shen R. Jiang Y. Xu W. Gu M. (2020). Identification of biomarkers predicting the chemotherapeutic outcomes of capecitabine and oxaliplatin in patients with gastric cancer. Oncol. Lett. 20, 290–291. 10.3892/ol.2020.12153 33029206 PMC7530885

[B74] Zheng L. Dai H. Zhou M. Li M. Singh P. Qiu J. (2007). Fen1 mutations result in autoimmunity, chronic inflammation and cancers. Nat. Med. 13, 812–819. 10.1038/nm1599 17589521

[B75] Zhou M. Wang B. Li H. Han J. Li A. Lu W. (2021). RNA‐binding protein SAMD4A inhibits breast tumor angiogenesis by modulating the balance of angiogenesis program. Cancer Sci. 112, 3835–3845. 10.1111/cas.15053 34219323 PMC8409301

[B76] Zou Y. Xu S. Xiao Y. Qiu Q. Shi M. Wang J. (2018). Long noncoding RNA LERFS negatively regulates rheumatoid synovial aggression and proliferation. J. Clin. Investigation 128, 4510–4524. 10.1172/JCI97965 PMC6159954 30198906

