# Supplementary figures and images for: Exploration of prognostic genes and risk signature in breast cancer patients based on RNA binding proteins associated with ferroptosis
Source: Front Genet. 2023 Feb 24;14:1025163. doi: 10.3389/fgene.2023.1025163 (PMC9998954; doi:10.3389/fgene.2023.1025163)

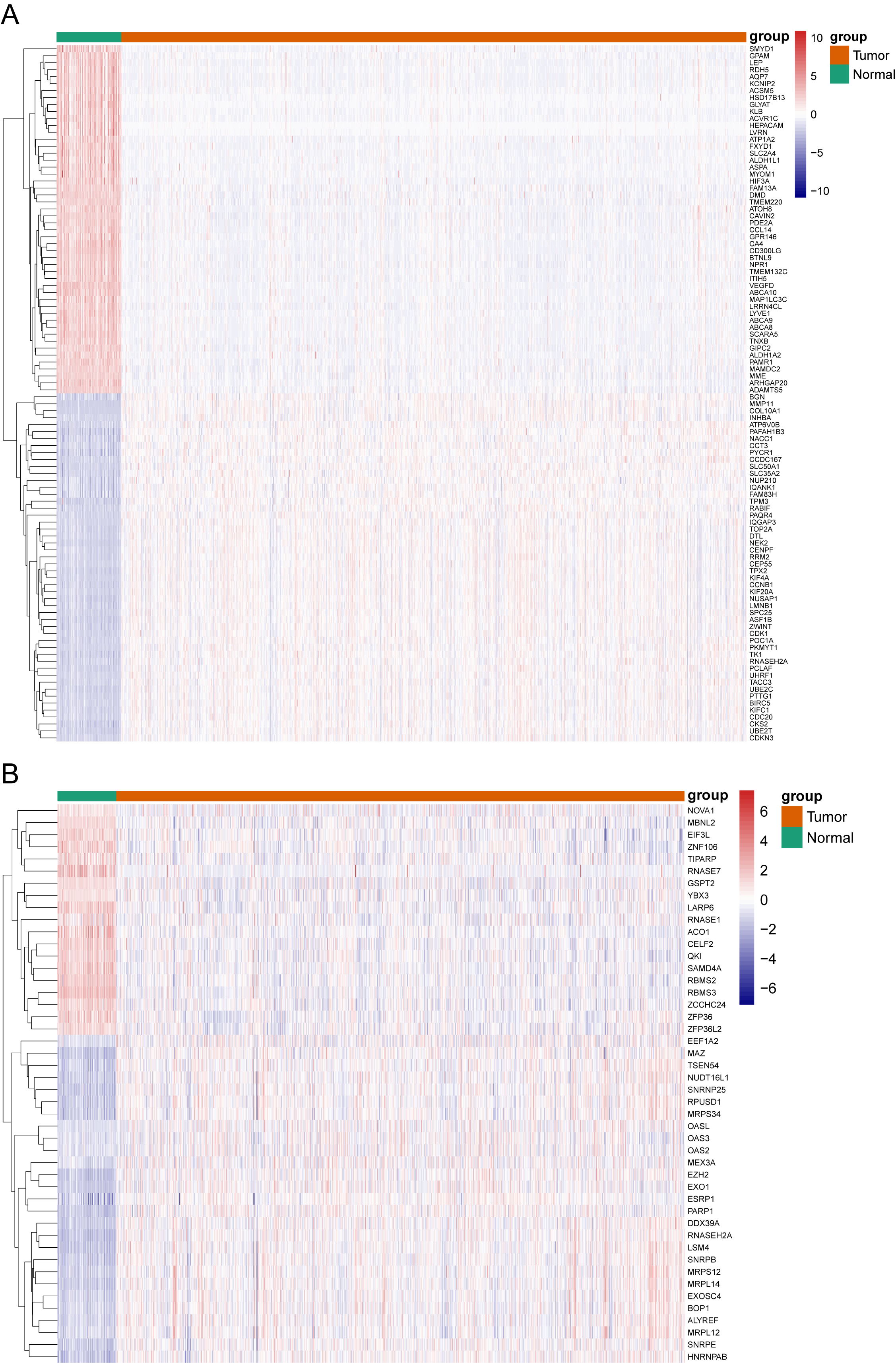

Supplement: Supplementary file 1 [file Image1.TIFF]

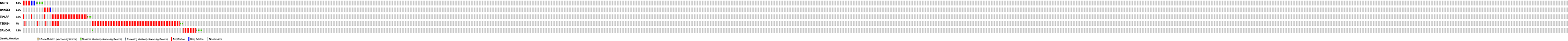

Supplement: Supplementary file 4 [file Image3.TIF]

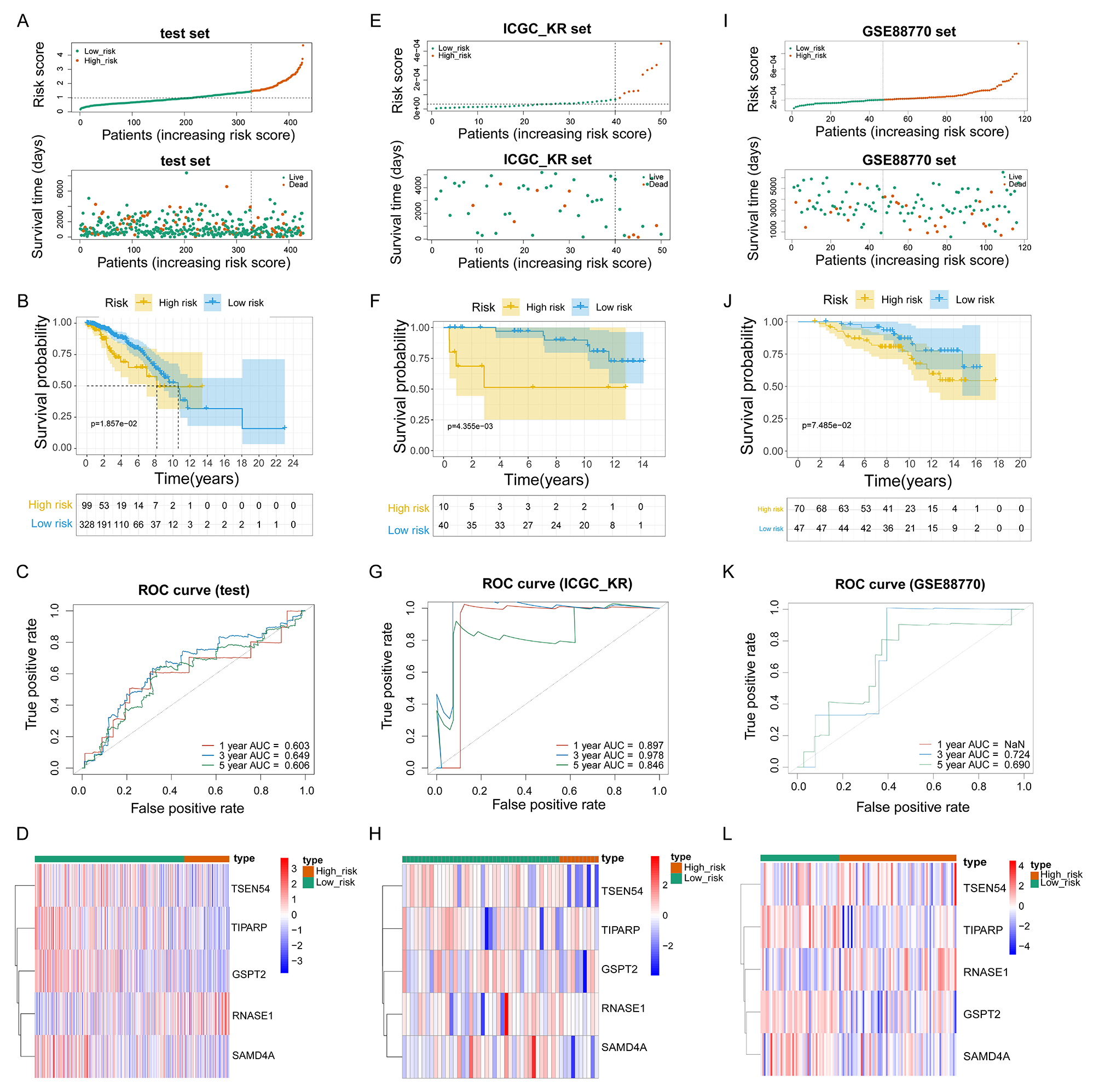

Supplement: Supplementary file 6 [file Image2.TIF]
